# Genome-wide detection of conservative site-specific recombination in bacteria

**DOI:** 10.1371/journal.pgen.1007332

**Published:** 2018-04-05

**Authors:** Ognjen Sekulovic, Elizabeth Mathias Garrett, Jacob Bourgeois, Rita Tamayo, Aimee Shen, Andrew Camilli

**Affiliations:** 1 Department of Molecular Biology and Microbiology, Tufts University School of Medicine, Boston, Massachusetts, United States of America; 2 Department of Microbiology and Immunology, The University of North Carolina at Chapel Hill, Chapel Hill, North Carolina, United States of America; Université Paris Descartes, INSERM U1001, FRANCE

## Abstract

The ability of clonal bacterial populations to generate genomic and phenotypic heterogeneity is thought to be of great importance for many commensal and pathogenic bacteria. One common mechanism contributing to diversity formation relies on the inversion of small genomic DNA segments in a process commonly referred to as conservative site-specific recombination. This phenomenon is known to occur in several bacterial lineages, however it remains notoriously difficult to identify due to the lack of conserved features. Here, we report an easy-to-implement method based on high-throughput paired-end sequencing for genome-wide detection of conservative site-specific recombination on a single-nucleotide level. We demonstrate the effectiveness of the method by successfully detecting several novel inversion sites in an epidemic isolate of the enteric pathogen *Clostridium difficile*. Using an experimental approach, we validate the inversion potential of all detected sites in *C*. *difficile* and quantify their prevalence during exponential and stationary growth *in vitro*. In addition, we demonstrate that the master recombinase RecV is responsible for the inversion of some but not all invertible sites. Using a fluorescent gene-reporter system, we show that at least one gene from a two-component system located next to an invertible site is expressed in an on-off mode reminiscent of phase variation. We further demonstrate the applicability of our method by mining 209 publicly available sequencing datasets and show that conservative site-specific recombination is common in the bacterial realm but appears to be absent in some lineages. Finally, we show that the gene content associated with the inversion sites is diverse and goes beyond traditionally described surface components. Overall, our method provides a robust platform for detection of conservative site-specific recombination in bacteria and opens a new avenue for global exploration of this important phenomenon.

## Author summary

Bacteria in many ecological niches experience a common challenge in the form of unpredictable environmental fluctuations. Rapid adaptation to challenging conditions is important for bacterial survival and successful proliferation. Altering gene expression through DNA inversion is a common mechanism adopted by many bacterial species that allows quick generation of distinct subpopulations with altered fitness. The characterization of these systems beyond a few classical cases is lagging due to the difficulties to accurately detect such inversion on a population level. In this study, we implement an easy-to-use method for detecting small genomic inversions in bacterial genomes. We successfully applied our approach to detect known and novel inversion sites in *C*. *difficile*. We further show that all detected sites undergo inversion and exist at different frequencies *in vitro*. The inversion of several sites seems dependent on the master recombinase RecV. We expand our analysis to a large collection of bacterial and archaeal strains and show that our method can be globally applied for detection of small genomic inversions. Taken together, this study advances the ability to characterize this important phenomenon.

## Introduction

Seemingly clonal microbial populations often exhibit phenotypic and behavioral heterogeneity. This feature is widely observed in the microscopic world–from viruses to protozoa–and reflects the common need to adapt to the ever-changing environmental milieu. While point mutations and horizontal gene transfer influence long-term microbial evolution, sudden environmental fluctuations leave little room for the comparatively slow, conventional gene-induction response to facilitate adaptation. A common bet-hedging strategy adopted by bacteria is to alter the expression of specific cellular components (e.g. flagella or pili) in a dichotomous on-off mode. This process, termed phase variation, is heritable, reversible, and occurs at frequencies well-above typical mutation rates [1]. In addition, phase variation is a stochastic process for generating distinct subpopulations that might have better fitness or survival rate under rapidly changing, challenging conditions. The degree of diversity solely depends on the number of phase-variable loci. Accordingly, phase variation can quickly become the major source of heterogeneity with a profound impact on the survival or fitness capacity of the population as a whole. This is best exemplified in pathogens that need to constantly change the expression of surface antigens as a consequence of the pressure from the host immune system [2–4].

The underlying sources of bacterial heterogeneity are fundamentally diverse. One common mechanism of phase variation involves site-specific DNA inversions, catalyzed by the action of one or multiple tyrosine or serine-type recombinases [5, 6]. The inverted genomic DNA fragment is frequently located in intergenic or 5’ untranslated regions and is delimited by short, inverted repeats. The inversion is typically *cis*-acting and has a direct impact on either the transcription or translation of the neighboring genes. This process creates a binary system that is either in a permissive (on-configuration) or non-permissive (off-configuration) state and as such is often dubbed a genetic or molecular switch. The net result is the creation of a heterogeneous bacterial community structure that is characterized by the co-existence of phenotypically distinct subpopulations. While switching is considered a stochastic process, environmental factors such as temperature, amino acids, carbon sources, osmolarity and iron limitation can influence inversion frequencies [7–10]. Additionally, an alternative model where cooperation rather than stochasticity is the driving force behind the switching has been proposed recently [11]. Recent evidence of lipoprotein phase variation in *Mycoplasma gallisepticum* and *M*. *agalactiae* also points towards a nonstochastic model during infection [12–14].

Phase variation as a result of DNA inversion was initially described in *Salmonella enterica* and *Escherichia coli* where it was associated with biphasic expression of flagella and pili respectively [15, 16]. Using an analogy-based approach, other bacterial species were also found to use similar DNA inversion-mediated phase-variable mechanisms to control the expression of pili and related cell-surface appendages [15, 17–21]. Expression of other surface components, such as outer membrane proteins and lipoproteins, have also been found to depend on DNA inversion [22–25]. However, DNA inversions do not affect merely surface components. For example, complex rearrangements among restriction-modification systems giving rise to differential methylation patterns have been extensively studied [26–31]. Additionally, DNA inversions are not confined to the bacterial realm, since they occur in bacteriophages, for example P1 and Mu, and plasmids such as R64 and p15B [32–34]. Finally, various non-surface components have been described to phase vary by mechanisms unrelated to DNA-inversion and ultimately influence multiple phenotypes related to immunoevasion, niche adaptation and virulence [35–42].

Despite the established importance and the substantial number of described examples in the literature, detection of novel DNA-inversion events remains a difficult task. This is primarily due to the lack of conserved and easily identifiable features specific to the inversion process. Site-specific recombinases cannot be used to determine the presence of genomic inversions in part because they are not intrinsically associated with the invertible site and might be encoded elsewhere in the genome. The only other hallmark of invertible sites are terminal inverted repeats. However, these are very short, typically in a range of 10–20 nucleotides and can often harbor mismatches that further hinders the ability of accurate detection.

Recent technical advances have enabled researchers to probe bacterial population diversity to unprecedented levels. The advent of affordable next-generation sequencing has proved to be specifically useful in this regard as deep sequencing of genomic DNA harvested from a population of cells allows the capture of associated genomic heterogeneity in its entirety, including small DNA-inversions [43, 44]. However, the majority of tools aimed at genomic structural variation discovery were developed for eukaryotic and specifically human genomes, especially in the context of cancer-associated chromosomal rearrangements [45, 46]. Recently, a workflow incorporating several previously developed tools was tested for structural variation detection in bacteria. While single-nucleotide polymorphisms, small insertions, deletions, duplications and translocations were identified with high sensitivity, inversions remained notoriously difficult to detect [47].

In this manuscript, we present a simple and easily applicable method for detection of conservative site-specific recombination events by whole-genome sequencing or from previously generated sequencing datasets. We first used simulated sequencing data to conceptually validate our approach and to optimize detection parameters. We then applied this method to an epidemic isolate of *Clostridium difficile*, for which we confirmed four known and identified three novel inversion sites. Using an experimental approach, we validated the inversion potential of all identified sites, quantified their prevalence in exponentially and stationary growth phase and assessed the DNA inversion dependence on the major recombinase, RecV. Furthermore, using a fluorescent-reporter assay, we show that one gene found next to the inversion site is expressed in a phase-variable manner. Finally, we extend our analysis to 209 bacterial and archaeal sequencing datasets and show that this method can be used to detect known and novel inversion sites.

## Results

### Global profiling of genomic inversions using high-throughput sequencing

**Fig 1:**
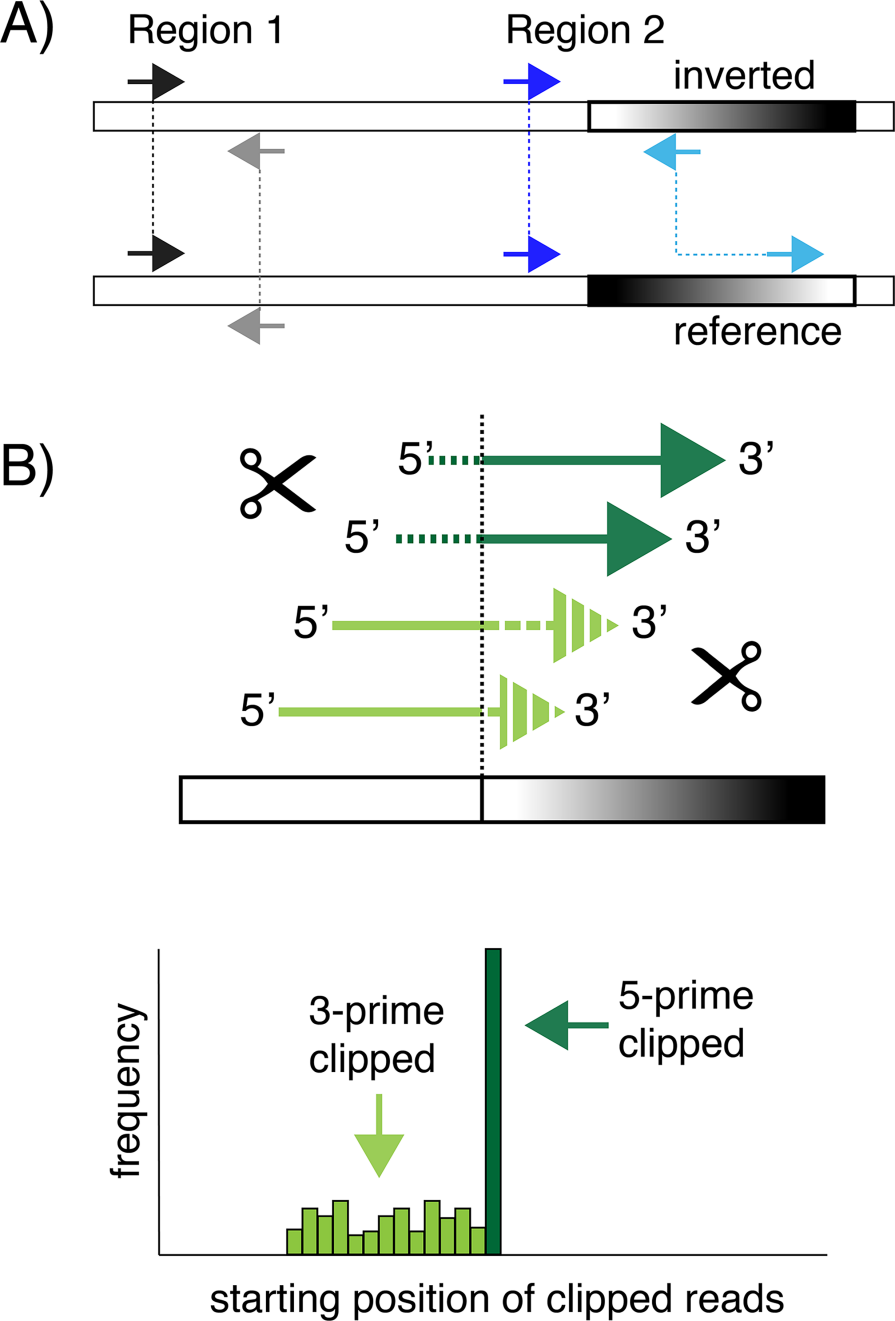
Genomic inversions are detected by clustering unusual read pairs. (A) DNA inversion results in read pairs with same relative orientation and increased inner-mate distance. The invariable DNA segment is illustrated by a white box and the invertible segment by a black-and-white gradient box. Individual reads are illustrated for two distinct regions by colored arrows. (B) Top cartoon: reads that overlap the junction of invertible sites are trimmed either on the 5-prime end (dark green) or 3-prime end (light green) during the alignment step. The trimmed portion of the reads are shown as dashed. Only the left side of the inversion site is illustrated. Bottom cartoon: histogram illustration of the frequency of starting position of all trimmed reads. Read trimmed at 5-prime end all start at the same genomic position and present high enrichment. Starting position of 3-prime end trimmed read is variable and present low enrichment.

We reasoned that genomic inversions could be detected by looking for specific signatures following high-throughput deep sequencing of microbial genomes. Typically, paired-end sequencing on Illumina’s platforms produces millions of intrinsically associated pairs of short sequences called reads. Upon mapping to the reference sequence, these pairs of reads are characterized by similar inner-mate distance and converging orientation, i.e. one read from a pair maps to the forward strand and the other read from a pair maps to the reverse strand of the reference sequence (region 1 in Fig 1A) [48]. An atypical orientation of paired reads is produced when an inverted segment, illustrated by the black-and-white gradient in Fig 1A, is sequenced and mapped back to the unchanged, reference sequence. The resulting read pairs have the same orientation, i.e. both reads map to the same strand. Additionally, the read pair has an increase in the inner-mate distance matching approximately the size of the inversion. This scenario is possible if one read from a pair maps to the invariable region of the genome and the other read from a pair maps to the invertible segment (region 2 in Fig 1A). Therefore, the presence of read pairs having the same orientation and higher mean inner-mate distance can be used as specific signature to detect genomic inversions.

In addition to identifying invertible regions, recombination sites at single-nucleotide level can also be inferred from genomic high-throughput sequencing. As illustrated in Fig 1B, individual reads that span the junctions of invertible sites are trimmed on either the 5-prime or 3-prime end by the alignment software during the mapping process. The trimming, also called clipping, allows for reads to be aligned despite not matching contiguously to the reference sequence. This process results in the accumulation of trimmed reads at both extremities of the invertible segment with the specific build-up of 5-prime end clipped reads at the site of recombination. Mapping the start position of the 5-prime clipped reads therefore allows for precise identification of the site where the DNA strand is broken by the enzymatic activity of the recombinase to initiate the strand exchange required for inversion.

### Successful detection of inversions in simulated sequencing datasets

We took an *in silico* approach to validate and optimize the proposed method. We first created an artificial and random DNA sequence that was subsequently used as the reference. We then manually reverse-complemented five distinct genomic regions ranging from 100 bp to 5,000 bp reproducing the natural process of conservative site-specific recombination. Next, both artificial genomes, i.e. reference and inverted, were individually used to simulate sequencing reads by emulating Illumina’s sequencing process with available bioinformatics tools [49]. In order to match variations in frequencies one could expect to find in invertible alleles in bacterial populations, we combined reads generated from both artificial genomes at proportions ranging from 0.01% to 50% (S1 Methods). Following read mapping to the reference genome, same-orientation reads as well as 5-prime end clipped reads were extracted from the dataset and analyzed to identify genomic locations with high enrichment indicative of putative inversions. As expected, all five genomic locations presented high enrichment for same-orientation and 5-prime end clipped reads perfectly matching the genomic locations where inversions were introduced (see S1 Fig for a representative example). These observations confirmed our initial hypothesis that small genomic inversions create specific and easily identifiable signatures in sequencing datasets.

We then sought to determine the optimal parameters under which inversions up to 5 kbp are detected. We found that small read lengths (e.g. 50 bp) and larger insert-sizes (e.g. ≥ 500 bp) allowed for better detection of inversion (S2 Fig). Additionally, read length was critical for detection of small inversions. For instance, no reads could be detected for a 100 bp inversion with read length of 150 bp regardless of other parameters. Finally, the limit of detection, arbitrarily defined as a cluster of ≥10 reads, was found to be significantly influenced by all three variables. Using optimal parameters (i.e. smaller read length and larger insert-size), the limit of detection (i.e. the frequency of the inversion in the population) was estimated to be 0.1% for inversions larger than 500 bp and approximately 1% for inversions smaller than 500 bp. For suboptimal conditions (i.e. longer read length and smaller insert-size), the limit of detection increased above 1% but was lower than 10% in all tested conditions (S2 Fig). The limit of detection was also directly proportional to the sequencing depth (S3 Fig). Taken together, these observations indicate that we can detect rare events of genomic inversions when a reference sequence and sufficient sequencing depth are available.

### Inversion signatures are present in the *Clostridium difficile* genome

Following the *in silico* validation of the method, we proceeded to determine the presence of genomic inversions in the opportunistic pathogen, *C*. *difficile*, where prior work has identified four short invertible regions designated “Cdi” (for *Clostridium difficile* inversion) [22, 50, 51]. Two of these invertible sites, Cdi1 and Cdi4, have been shown to control the expression of adjacent genes (*cwpV* and flagellar, respectively) in a phase-variable manner. Cdi2 and Cdi3 are predicted invertible sites that have not been experimentally confirmed [50]. In order to determine if additional and similar genetic switches were present within *C*. *difficile* genomic context, we took advantage of existing datasets deposited in Sequence Read Archive repository (SRA, https://www.ncbi.nlm.nih.gov/sra). Among the 8,468 results for *C*. *difficile*, we chose a single dataset derived from the sequencing of an epidemic ribotype-027 isolate at The Wellcome Trust Sanger Institute. We chose this dataset mainly because of its high sequencing depth (93,569,362 paired reads of 75 bp), which ensured that we could identify, in theory, inversions present at frequencies down to 0.1%. Reads were mapped to the *C*. *difficile* R20291 reference genome with high efficiency (90,185,223 mapped reads, 96.4%) resulting in mean coverage of 1614X. Extraction of reads with the same relative orientation yielded a total of 256,856 pairs (513,712 individual reads, 0.57%), while extraction of 5-prime end clipped reads returned a total of 37,062 (0.04%) reads. These low counts are consistent with the premise that unidirectional and 5-prime end clipped reads are rare.

**Fig 2:**
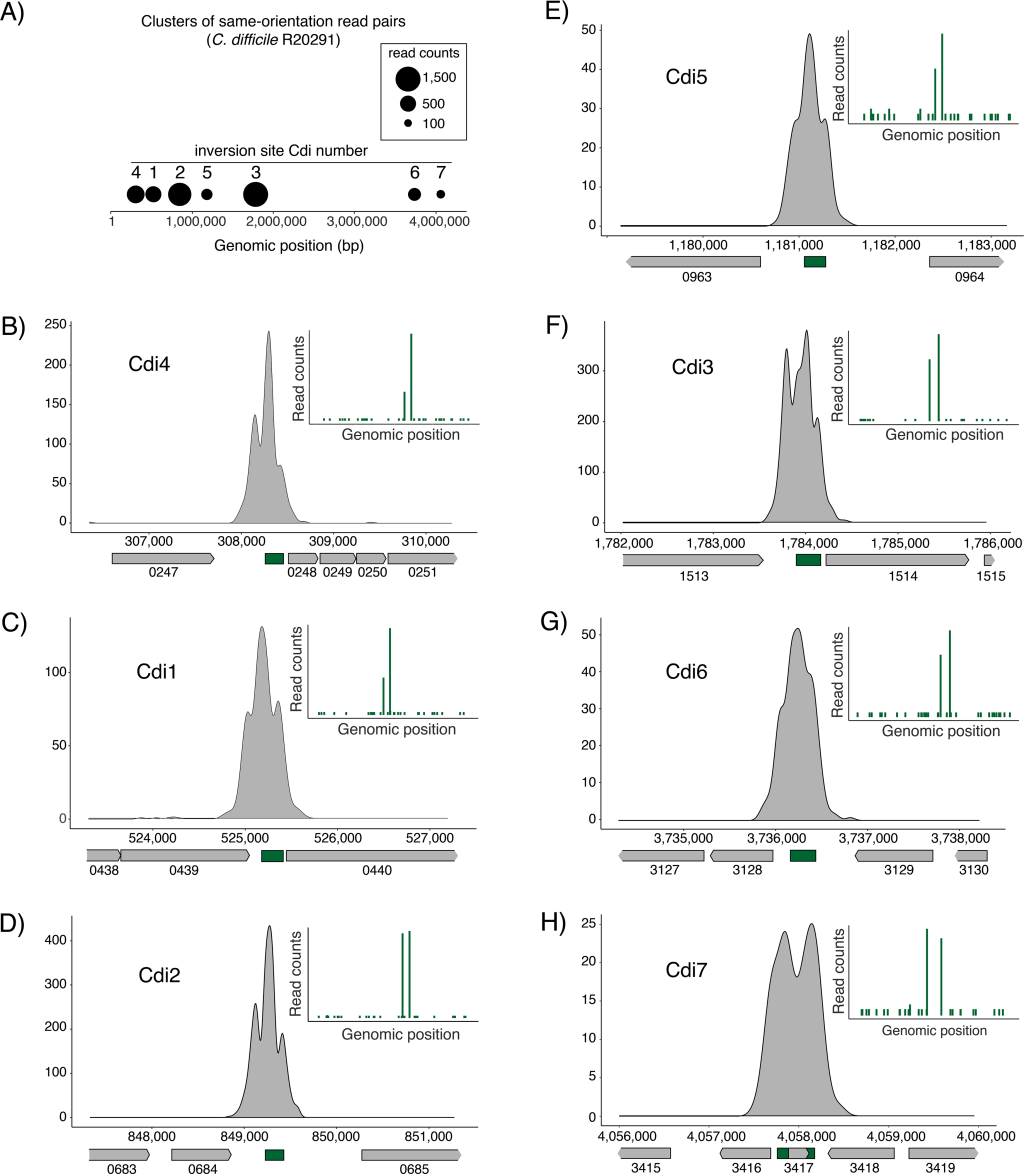
*C*. *difficile* R20291 genome harbors seven inversion sites. (A) Seven distinct clusters (black circles) composed of unidirectional read pairs are detected in *C*. *difficile* R20291 genome. Each cluster is numbered following a previously described convention. (B-H) Illustration of unidirectional reads enrichment relative to the genomic on-scale context. The *y* axis represents read number and the *x* axis represent genomic position. Associated open reading frames are shown with arrowed boxes and labeled by the numeric value from their respective locus_tag number. The position of the invertible segment is illustrated by the dark green box relative to the gene content. The enrichment of 5-prime end clipped reads is shown in the separate plot at the top right corner. The two major dark green bands correspond to left and right boundary of the invertible site.

**Fig 3:**
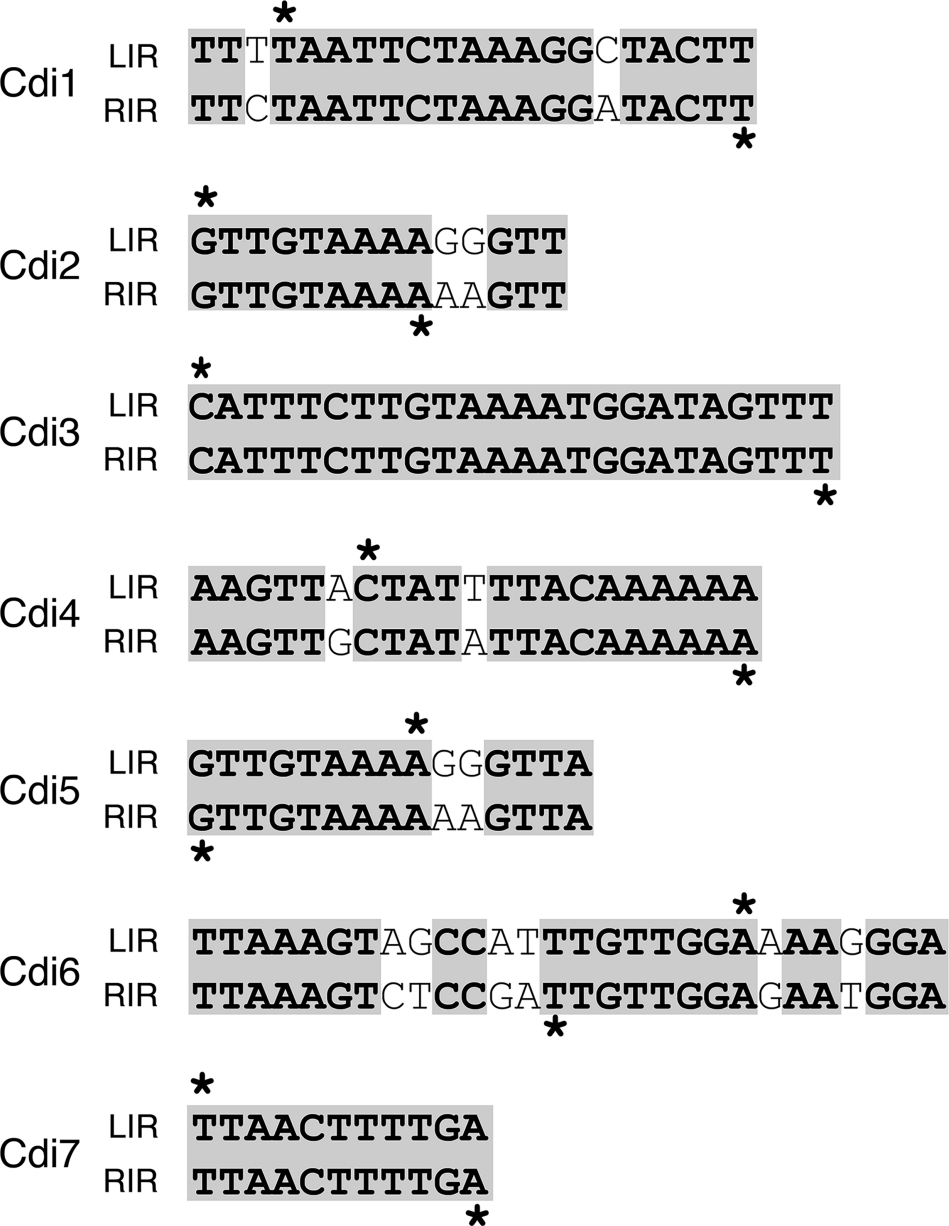
Terminal inverted DNA repeats border inversion sites in *C*. *difficile*. LIR stands for “left inverted repeat” and RIR stands for “right inverted repeat”. Gray shading illustrates sequence homology. The asterisk (*) points to the nucleotide where recombination is taking place as identified by enrichment of 5-prime end clipped reads.

Cluster analysis revealed seven distinct groups with inversion sizes ≤ 5 kbp and variable read counts (Fig 2A). Close examination of six individual clusters revealed strong enrichment of unidirectional read pairs in intergenic regions (Fig 2B–2G), while one cluster spanned a small open reading frame (Fig 2H). All identified regions also had a sharp accumulation of 5-prime end clipped reads on two distinct genomic positions, effectively identifying putative inversion boundaries (Fig 2B–2H, separate plot in the top right corner, green bars). Since inverted repeats border the putative inversion segments, these regions may be recombining to generate an inversion event (Fig 3). Additionally, Cdi2 and Cdi5 sites are bordered by identical inverted repeats, suggesting recognition by a common recombinase. All other inverted repeats were unrelated.

In the order of appearance, the first two clusters correspond to the previously described flagellar (Cdi4) and *cwpV* (Cdi1) switches respectively. Corresponding left and right inverted repeats as well as the size of the inversion perfectly match with previous reports [22, 51]. The third cluster correspond to the previously cited Cdi2 [50], located in vicinity of a signaling protein (locus_tag CDR20291_0685) with conserved diguanylate cyclase (GGDEF), diguanylate phosphodiesterase (EAL), and sensor PAS domains. In an earlier study, the GGDEF-domain was shown to be defective but the EAL-domain was found intact [52]. An almost identical element bordered by the same inverted repeats is part of the fourth cluster named Cdi5. CDR20291_0963 is the first gene in an apparent operon of three encoding a putative membrane bound O-acetyl transferase. The remaining coding sequences from the operon, CDR20291_0962 and 0961 encode respectively a SGNH hydrolase-like protein and a hypothetical protein with no conserved domains. The fifth cluster corresponds to the previously cited Cdi3 [50], located upstream of another putative signaling protein (locus_tag CDR20291_1514) containing both GGDEF and EAL domains. Once again, the GGDEF domain is mutated [52], indicating that this protein is likely involved in degradation rather than formation of c-di-GMP. The sixth cluster, termed Cdi6, is located upstream of an apparent operon of three genes encoding a classical two-component regulatory system. CDR20291_3127 encodes a putative signal transduction histidine kinase that carries a dimerization/phosphoacceptor domain, a histidine kinase domain on the C-terminal cytosolic portion, and a galactose binding-like domain on the N-terminal extra-cytoplasmic portion. This histidine kinase gene is flanked by genes encoding two cognate response regulators, CDR20291_3126 and 3128, which have conserved C-terminal effector and N-terminal receiver domains. The seventh cluster, Cdi7, spans a small open reading frame of 228 nucleotides encoding a hypothetical protein (locus_tag CDR20291_3417) containing a domain of unknown function DUF1413. Collectively, these results suggest that there are more invertible sites in the *C*. *difficile* R20291 genome than previously thought; these sites may influence a variety of cellular functions based on the functional diversity of genes encoded in vicinity.

### All identified regions undergo inversion in *C*. *difficile*

**Fig 4:**
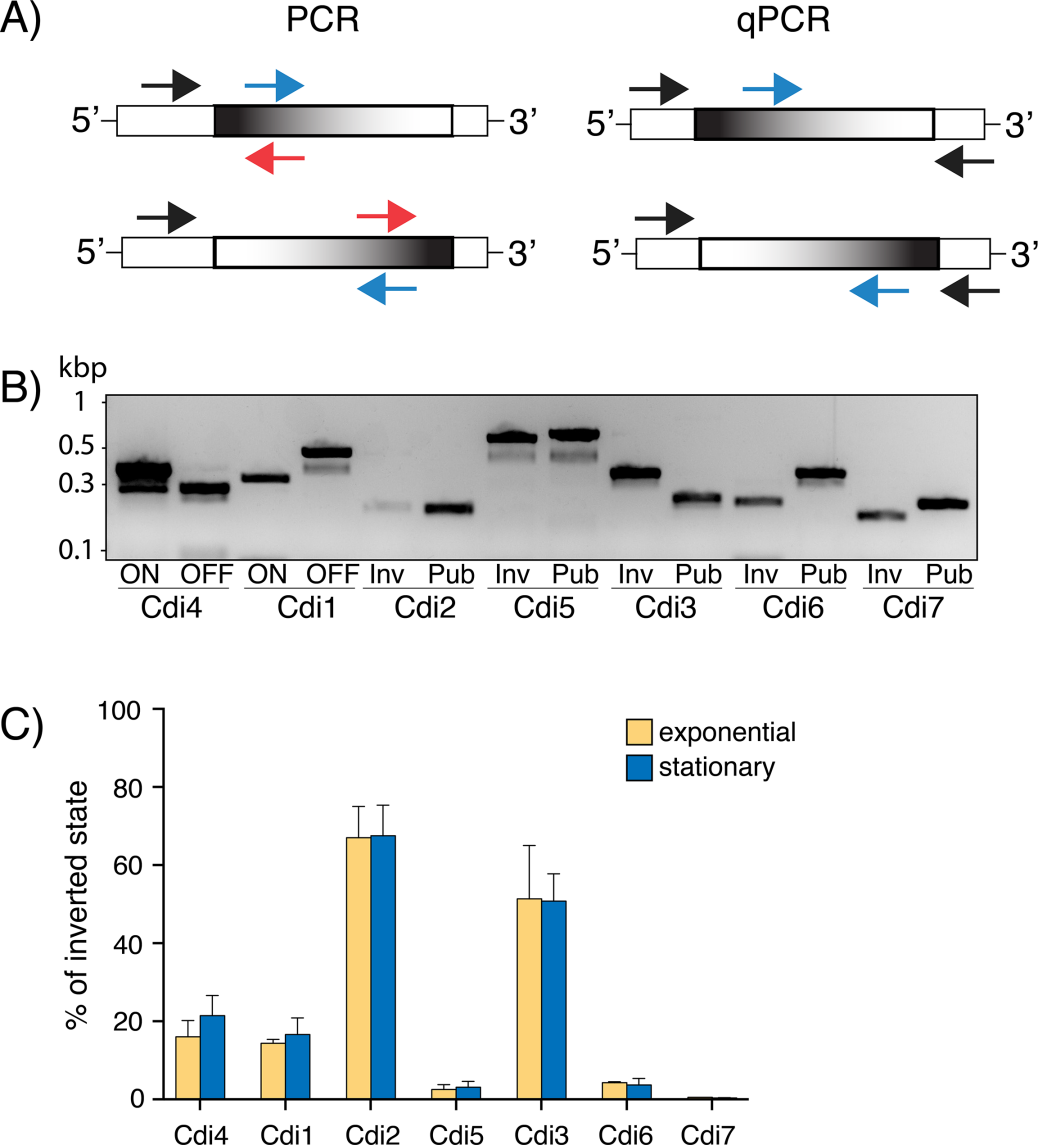
Detection and quantification of inversion events in *C*. *difficile*. (A) Schematic representation of orientation-specific PCR and qPCR strategies. Invariable DNA region is illustrated by white and invertible region by black-and-white gradient box. Primers used for PCR amplification are shown as arrows. (B) The agarose gel showing PCR products for either published (Pub) or inverted (Inv) orientation. For Cdi4 and Cdi1, permissive (ON) and restrictive (OFF) orientations for downstream gene expression have been determined elsewhere. DNA from 3 biological replicates was tested and one representative example is shown. (C) Quantification of inverted orientation relative to the published orientation by qPCR. Presented are means from 3 biological replicates. Error bars correspond to 2 standard deviations.

To determine if identified regions are subject to inversion during bacterial growth, we performed orientation-specific PCR on genomic DNA extracted from exponentially growing cells. Our PCR strategy and primer design are presented in Fig 4A. Using this approach, we detected two bands with the expected molecular weight corresponding to both orientations for all seven identified regions (Fig 4B). We refer to a “published” orientation to indicate the sequence present in the published genome of *C*. *difficile* R20291, and “inverse” orientation to refer to a sequence with an inversion in the corresponding site. Orientations were confirmed by DNA sequencing of the PCR products. This result effectively confirmed that all invertible segments, identified with our bioinformatics approach, exist in both orientations in actively growing cells under normal lab conditions.

Since our end-point PCR approach did not allow for quantification of the frequency at which different orientations exist within bacterial population, we used new primers with high and very similar amplification efficiencies to allow for relative quantification using the ∆∆C_T_ method (S2 Methods). The percentage of the inverted state relative to the published orientation for *C*. *difficile* R20291 for all seven switches was determined during mid-exponential and early-stationary phase growth rate (Fig 4C). As expected for a stochastic process, we found that different switches coexist in different proportions. In line with previous findings [51], the flagellar switch, Cdi4, occurs predominantly in the published (ON) orientation as the mean frequency of the inverted (OFF) state was found to be 16.0%, 12.6–20.2% (2 Standard Deviations or 95.45% confidence levels) in actively growing cells. Similarly, the percentage of the inverted state (ON) was 14.3%, 13.4–15.4% for the *cwpV* switch, Cdi1, which is marginally higher than previously reported values (~5–10%) [22, 53]. Switches detected upstream of both cyclic-di-GMP signaling proteins, namely Cdi2 and Cdi3, were found to have higher inverted orientation proportions with respective frequencies of 67.0%, 57.8–75.1% and 51.4%, 37.7–65.0%. In contrast, the remaining three switches inverted at low frequency. Cdi7 had the lowest proportion quantified at 0.5%, 0.4–0.6% followed by Cdi5 at 2.6%, 1.7–3.8% and Cdi6 at 4.3%, 4.1–4.6%. Substantial differences in orientation frequencies suggest a low level of co-regulation, which in turn implies that *C*. *difficile* exhibit remarkable variations in inversion orientations on a single-cell level. Predictably, this should increase overall population diversity and collective functions. However, this presumed diversity is relatively stable, as nearly identical inversion proportions were found when genomic DNA was harvested from cells grown to early stationary growth phase. Collectively, these results demonstrated that our method allows for accurate and genome-wide detection of phase variable-like genomic inversions with wide range of input frequencies.

### Expression of CDR20291_3128 is consistent with phase variation

**Fig 5:**
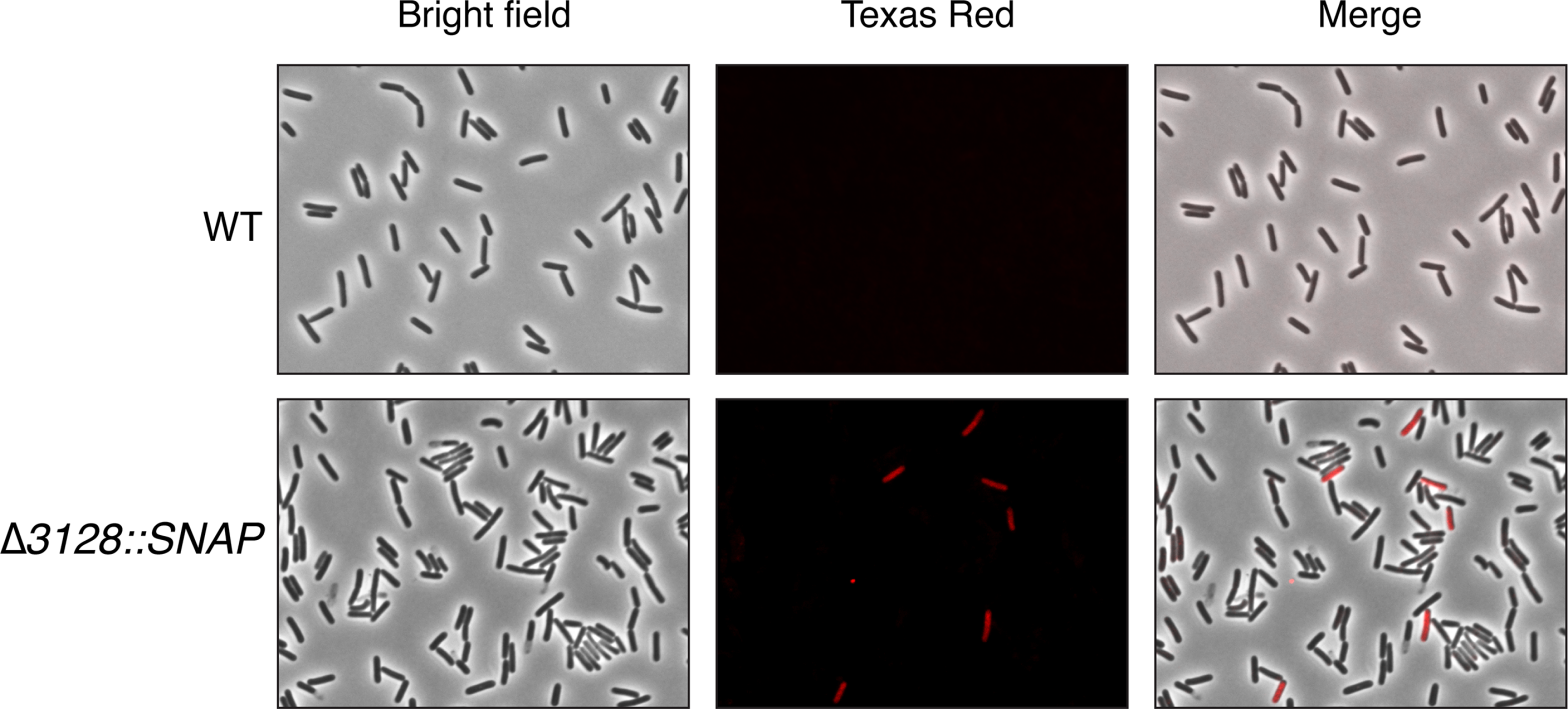
Expression of CDR20291_3128 is consistent with phase variation. Liquid culture samples from *C*. *difficile* R20291 and ∆3128::SNAP-tag were analyzed by light and fluorescence microscopy following staining with SNAP-Cell TMR-Star substrate. The left column shows the view of total cells in bright field light (BF), the middle column shows the SNAP-tag expressing cells pseudocolored in red, and the right column shows the merged pictures of BF and fluorescence.

Since the previously characterized *C*. *difficile* switches, Cdi1 and Cdi4, have been shown to control the expression of downstream genes in the classical on-off mode reminiscent of phase variation, we sought to determine if genes found in the vicinity of newly identified switches might experience the same type of expression control. We thus developed a strategy to detect expression of CDR20291_3128 on a single-cell level using a fluorescent transcriptional reporter. Analysis of previously published transcriptomic data suggested that CDR20291_3128 is part of an operon with CDR20291_3127 and 3126 (S4 Fig) [53]. We thus decided to replace the entire coding sequence of the first gene from the predicted operon, CDR20291_3128, with a *C*. *difficile* codon-optimized *SNAP-tag* gene reporter. The reporter strain, *C*. *difficile* R20291 ∆*3128*::*SNAP-tag*, was labeled with cell-permeable substrate and observed under a fluorescence microscope. As expected, no fluorescent signal was observed for the wild type R20291 strain as it lacks the SNAP-tag reporter. On the other hand, ∆*3128*::*SNAP-tag* cells displayed a robust but bimodal signal consistent with the hypothesis that the expression of CDR20291_3128 is likely phase-variable (Fig 5). Counts of bright cells were done on 15 images from different fields from two biological replicates and compared to the counts of total number of cells (~300 cells / image) using ImageJ. The proportion of fluorescent-positive cells was estimated to be 4.28% ± 2.15% (standard deviation) which is virtually identical to the inverted orientation of the switch as quantified by qPCR (4.3%, 4.1–4.6%, Fig 4C). This suggests that the Cdi6 might be directly regulating the expression of the operon in an on-off manner as it was previously demonstrated with Cdi4 and Cdi1 switches. Accordingly, the inverted orientation likely represents the on-configuration and the published orientation represents the off-configuration of the switch.

### RecV is required for inversion of some but not all identified switches

**Fig 6:**
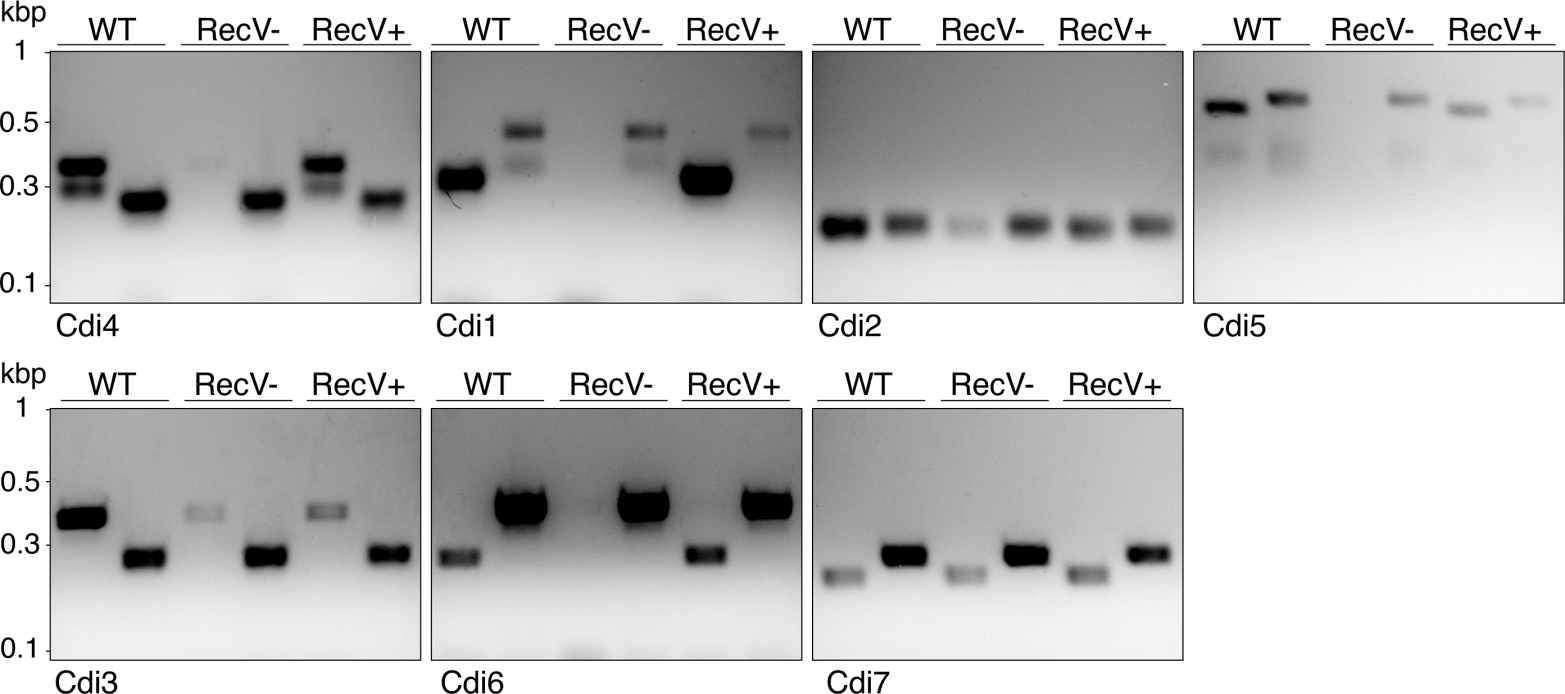
Impact of *recV* loss on orientation of invertible sites in *C*. *difficile*. Agarose gels showing PCR products from published (Pub) and inverted (Inv) orientations in the wild type (WT), the *recV* null mutant (RecV-) and following complementation from a plasmid (RecV+).

The tyrosine recombinase RecV catalyzes inversion of flagellar (Cdi4) and *cwpV* (Cdi1) switches [22, 51], so we sought to determine if RecV is also necessary for the inversion of newly identified switches. Inactivation of *recV* should prevent further inversions, locking individual switches in either the published (pub) or inverted (inv) orientations. We performed orientation-specific PCR in R20291 *recV*^−^(*recV*::*ermB*) strain constructed using ClosTron mutagenesis [53]. Consistent with previous reports, we observed a single orientation for both flagellar (Cdi4, inverted) and *cwpV* (Cdi1, published) switches in the *recV*^−^strain (Fig 6). Similarly, we observed only published orientation for Cdi5 and Cdi6 switches in the *recV*^−^background. On the other hand, we observed both orientations for Cdi2 and Cdi3 albeit with different band intensities as compared to the wildtype strain suggesting impaired but not complete loss of inversion. Finally, we did not detect any alterations for Cdi7 in the *recV*^−^background. However, due to the non-quantitative aspect of end-point PCR, we could not draw conclusions based merely on band intensities. We therefore used a more sensitive qPCR for the following experiments.

**Fig 7:**
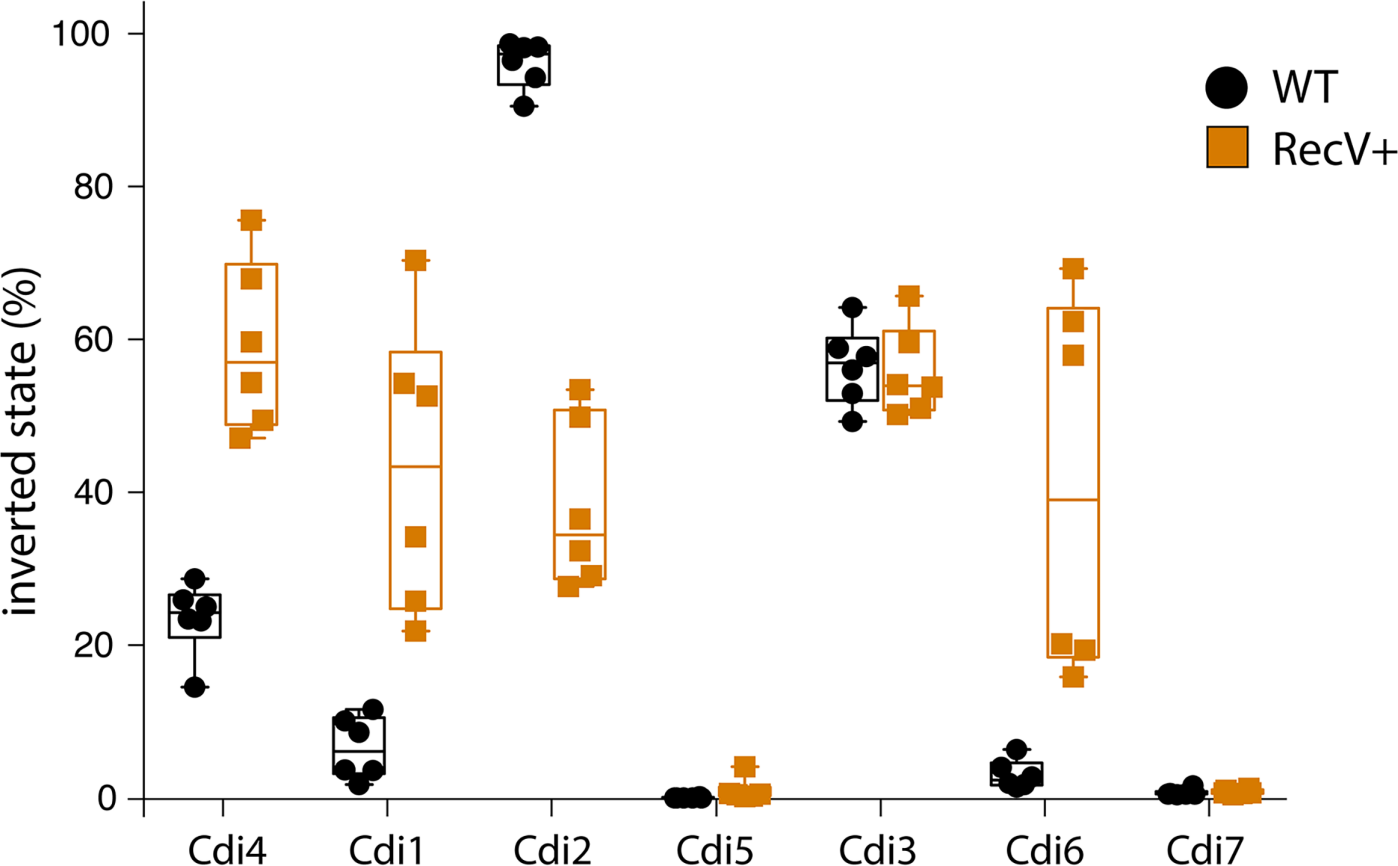
Impact of *recV* overexpression on the orientation frequency in *C*. *difficile*. *recV* was overexpressed from a plasmid in the wild type background. The *y* axis represents the percentage of inverted orientation relative to published orientation as determined in the wild type context carrying the empty pRPF185 plasmid (black filled circles) and during *recV* overexpression (orange filled squares) by qPCR. Reported are medians, upper and lower quartiles and extremes.

As a second approach, we reasoned that overexpression of RecV would disrupt the relative stoichiometry and cause shifting of baseline proportions of the distinct switch orientations. We therefore placed *recV* with its native Shine-Dalgarno sequence under the control of a tetracycline-inducible promoter. The resulting plasmid was transferred in *C*. *difficile* R20291 wildtype strain by conjugation. Following induction, genomic DNA was extracted and the relative proportions of all switches quantified by qPCR (Fig 7). Change in the inversion proportions when *recV* was overexpressed relative to wildtype strain carrying the empty plasmid was observed for Cdi4 and Cdi1 as expected, but also for Cdi2 and Cdi6. This is in agreement with our previous experiments in *recV*^−^background, as these switches were either locked (Cdi4, Cdi1 and Cdi6) or partially locked (Cdi2). No change in relative proportions was observed for Cdi5, Cdi3 and Cdi7 switches. This observation partially contrasts with our previous experiment where loss of *recV* significantly impacted both Cdi5 and Cdi3 inversion. Lastly, overexpression of RecV did not affect the frequency of Cdi7 switching. Taken together, these combined observations suggest that RecV is essential for inverting Cdi4 (flagellar switch), Cdi1 (*cwpV* switch) and Cdi6, partially required for inverting Cdi2, Cdi5 and Cdi3, and dispensable for inverting Cdi7. Thus, additional recombinases likely mediate inversion of some sites, either alone or in concert with RecV.

### Inversions are detected in a wide range of bacterial species

Having successfully detected known and novel invertible sites in *C*. *difficile*, we aimed to determine whether other bacterial species harbor similar inversion signatures. Once again, we took advantage of sequencing datasets deposited in Sequence Read Archive (SRA) and European Nucleotide Archive (ENA, https://www.ebi.ac.uk/ena)). We limited our analysis to a subset of available datasets satisfying the following criteria: minimum of 2 million read pairs, maximum read length of 150 bp and inner-mate distance lower than 1,000 bp. Preference was given to those datasets for which the full-length reference sequence was also available. Out of 209 analyzed sequencing datasets, 203 were of bacterial and 6 of archaeal origin (S2 Table). Bacterial samples were distributed among 13 phyla with marked bias toward Proteobacteria (n = 80) and Firmicutes (n = 66), reflecting the limited diversity of available sequences in SRA and ENA repository databases. However, the overall diversity was relatively high with 127 different genera encompassing 177 different species. The remaining 26 samples represented distinct subspecies, strains or serovars. Archaeal samples were from 2 phyla and represented 6 different genera and species. The analysis procedure was automated with a custom script and putative inversions were further manually scored based on the size, intergenic location and the presence of inverted repeats or recombinases in vicinity. A complete summary of the analysis is reported in S2 Table. Overall, we found clear signatures of inversions in 43 samples covering 30 different genera and 34 distinct bacterial species. No signs of inversions were found in our small subset of archaeal genomes. Putative inversions were found in between 10–50% of species within the bacterial phyla; Firmicutes (15/66, 23% of unique species), Bacteroidetes (7/20, 35%), Proteobacteria (8/80, 10%), Actinobacteria (3/19, 16%), Spirochaetes (1/8, 13%) and Synergistetes (1/2, 50%). Some interesting patterns were observed. Among 15 signature-positive unique samples from Firmicutes phylum, 14 belong to the class Clostridia and only 1 belong to the class Bacilli. Similarly, in the phylum Proteobacteria, the majority of signature-positive samples were detected in Gammaproteobacteria class (7/55, 13%), one in Betaproteobacteria (1/13, 8%) and none in Alphaproteobacteria (0/4), Deltaproteobacteria (0/2) and Epsilonproteobacteria (0/6). On the other hand, signature-positive samples were more evenly distributed in Bacteroidetes phylum (n = 20), with most positives in Bacteroidia class (5/8, 63%), one in Flavobacteriia (1/5, 40%) and Cytophagia (1/3, 50%) and none in Chitinophagia (0/2) and Sphingobacteria (0/2). Overall, we found that many bacterial species harbor signatures of inversion yet they appear to segregate in specific lineages. This observation hints at possible evolutionary conservation and selective pressures, but the small and uneven sample size hinders robust statistical analysis.

### Genes encoding diverse functions are found in vicinity of novel invertible sites

While DNA inversions have been readily described among bacteria, bacteriophages and plasmids, the diversity of the associated gene content was found to be relatively limited. Following identification of invertible sites among 34 distinct bacterial species, we proceeded to determine the surrounding genomic context. Unsurprisingly, we found high prevalence of recombination in between *hsdS* genes associated with type I restriction-modification (R-M) systems. This well-known phenomenon, described in a variety of bacterial species, is believed to influence global gene expression via alterations in methylation profiles (recently reviewed in [54]). We found signs of DNA inversions among type I R-M systems in 13 distinct bacterial species confirming their broad occurrence. Genes encoding cell surface structures such as fimbriae, flagella and pili were also prevalent in vicinity of invertible sites. Some of the identified invertible segments were previously described, such as the *fim* switch in *E*. *coli* and the *mad* switch in *Photorhabdus luminescens*, but others were unknown. For example, we found a novel invertible site in vicinity of fimbriae locus in *Providencia stuartii*. In contrast to *E*. *coli* and *P*. *luminescens* fimbriae switches that are short (296 bp and 266 bp respectively excluding inverted repeats) and contain only a promoter, this putative invertible segment is significantly larger (844 bp excluding 14 bp imperfect inverted repeats) and encloses an open reading frame encoding a recombinase.

In contrast to other clades, the most prevalent functions encoded in vicinity of invertible sites in Clostridia were related to intracellular signaling and chemotaxis (S2 Table). To the best of our knowledge, none of these invertible sites have been previously described. For example, *Anaerotignum propionicum* DSM 1682 (formerly *Clostridium propionicum*) harbors an inversion signature located in vicinity of a methyl-accepting chemotaxis protein. Similarly, another inversion signature is found in *Desulfitobacterium dehalogenans* ATCC 51507 next to an open reading frame encoding a putative signaling protein with a phosphohydrolase conserved HD-type phosphodiesterase domain. Similarly, two distinct invertible sites, both found in proximity of genes encoding chemotaxis components are present in *Clostridium thermocellum* DSM 1313. DNA inversion among prophage regions were readily detected in 4 species of Proteobacteria. The specific prophage regions seemed to revolve around structural genes, particularly tail fibers, which is also a well-known phenomenon occurring in bacteriophages such as P1 and Mu [33]. Finally, several species from Bacteroidetes phylum presented multiple DNA inversions spanning various cell surface components such as capsule and putative carbohydrate-binding receptors. This is consistent with previous reports where numerous invertible segments were found in several *Bacteroides* genomes [55, 56]. In summary, the association of identified invertible sites with genes encoded in their vicinity hints at possible *cis*-regulation of a diverse set of bacterial functions.

## Discussion

Small genomic inversions are known to be important mediators of gene expression in several bacterial species, but their systematic identification has been challenging. In this study, we developed a method for genome-wide detection of conservative site-specific recombination in bacteria using paired-end high-throughput sequencing. We demonstrate the utility of our method by detecting and experimentally confirming known and novel invertible sites in the opportunistic pathogen *C*. *difficile*. We also expand our analysis on a large collection of other bacterial species showing that both known and putative novel inversions can be readily detected with our approach.

### Paired-end sequencing can be used to detect inversions in bacterial genomes

While the use of next generation sequencing for detecting genomic structural variations has revealed genomic rearrangements such as deletions, insertions, inversions, duplications and translocations [44, 57], most of available tools have been designed for analyses of eukaryotic genomes. The drawback is that these tools often implement computationally demanding algorithms and do not always perform optimally on prokaryotic genomes. Recently, two attempts have been made to detect structural variation in prokaryotes. One method did not explicitly support inversion detection [58] and the other was applied only on a few selected *E*. *coli* species where *fim* switch and prophage shuffling was detected [59]. We reasoned that small inversions could be easily identified in bacterial genomes simply by clustering unusual paired reads that have the same relative orientation or 5-prime clipping (Fig 1). Using *in silico* simulations, we show that our approach is fairly flexible in terms of sequencing parameters allowing for previously generated datasets to be analyzed *a posteriori*. Furthermore, as reads smaller then 150 bp continue to be preferred in novel implementations of short-read sequencers (e.g. Illumina’s HiSeq X, HiSeq 3000, HiSeq 6000, NovaSeq600 and NextSeq), our method of genome analysis should stay relevant in the near term.

In theory, our method allows for global detection of genomic inversions regardless of the inversion size. However, large genomic inversions usually arise following homologous recombination in between substantial stretches of sequence homology scattered within the genome such as rRNA operons and transposable elements [60–62]. Because our method discards any reads that can equally map at multiple locations (which reduces false-positive calls), these large inversions are not detected. This can be overcome by using mate-pair libraries that allow for distant genomic regions to be joined and sequenced on short fragments producing insert sizes of several kilo base pairs. One potential drawback of our approach is the need for deep sequencing coverage to successfully detect rare inversion events. On the other hand, the method is simple to implement, does not require powerful computing capacity and uses only free and widely available tools for mapping and analysis.

### *C*. *difficile* genomes harbor numerous invertible segments

Previous work had identified four small genomic segments that undergo inversion in *C*. *difficile*. The putative inversion sites, termed Cdi1, Cdi2 and Cdi3, were identified in a global comparative genomics survey of three complete *C*. *difficile* genomes [50]. Cdi1 undergoes inversion and regulates the expression of the downstream *cwpV* gene in a phase-variable manner [22]. The remaining two inversion sites, Cdi2 and Cdi3, were never experimentally confirmed. An additional inversion site (Cdi4) was recently identified and shown to control the expression of flagella and toxins in phase-variable manner [51]. Using our paired-end analysis approach, we detected all four known invertible sites and identified three novel sites. The invertible sites detected are of similar size ranging from 154 bp to 230 bp; all sites occur within intergenic regions with the exception of Cdi7, which contains an open reading frame of 75 amino acids. This is consistent with previously characterized invertible segments. For example, *E*. *coli* (*fim* switch, 314 bp; *fot* switch, 312 bp), *Proteus mirabilis* (*mrp* switch, 251 bp), *P*. *luminescens* (*mad* switch, 257 bp) and *Klebsiella pneumoniae* (Kpc fimbriae switch, 302 bp) have small intergenic invertible segments with the exception of *Salmonella* whose flagellar switch is somewhat larger (966 bp) [15–18, 20].

While we experimentally confirmed that these putative sites undergo inversion (Fig 4B), we observed that the proportion for each state, i.e. inverted versus published orientation, was variable. This variability might reflect the complex nature of regulation in terms of required recombinases or selective fitness in our growth conditions. Regardless, switching frequency was independent of bacterial growth phase (Fig 4C). This is in sharp contrast to the *fim* switch regulation in both *E*. *coli* and *K*. *pneumoniae*. For example, transcription of *fimA*, the first gene downstream of the *fim* switch in *E*. *coli*, was found to be repressed as cells enter stationary phase in a manner dependent on the stationary phase-specific sigma factor, RpoS [63]. In *K*. *pneumoniae*, the *fim* switch is in the off-configuration during exponential growth in broth but undergoes inversion in the late stationary phase [21]. In contrast, the ratio of phase 1 versus phase 2 flagellar types in various *Salmonella* strains is mostly independent of growth phase [64], similar to *C*. *difficile* strain R20291 flagellar on and off isolates were found to remain stable in either exponential or stationary growth phases [51]. Interestingly, *flgB*, *cwpV*, both c-di-GMP regulators and the two-component system are part of the *agr* quorum-sensing regulatory network in *C*. *difficile* [65]. Thus, it is possible that the control of phase-variable genes is under the influence of cell density. Alternatively, other factors independent of growth phase might impact inversion proportions in *C*. *difficile* as it was shown for *E*. *coli* [7].

Small DNA inversions in bacterial genomes are commonly associated with phase-variable expression of neighboring genes. Two previously characterized switches in *C*. *difficile*, Cdi1 and Cdi4, also control the expression of their respective downstream genes in a phase-variable manner. The additional invertible sites identified in our study, namely Cdi2-3 and Cdi5-7, may similarly regulate the expression of their adjacent genes. Indeed, we determined that the first gene from the two-component system found downstream of the Cdi6 is likely expressed in a phase-variable manner (Fig 5) at levels similar to those detected in the qPCR orientation assay. However, assessing the direct impact of switch orientation on gene expression requires locking of the switch and measuring gene expression accordingly. Alternatively, restriction analysis of fluorescently labeled PCR products (GeneScan analysis) could be used [28]. Subsequent analyses are also needed to determine how switching alters gene expression, for example whether it modulates promoter orientation or affects transcriptional elongation.

The detection of invertible DNA sequences raises questions regarding the enzymes responsible for these inversions. Two evolutionarily and mechanistically distinct families of enzymes, namely serine and tyrosine recombinases [5], typically catalyze site-specific DNA inversions. In the majority of characterized systems, recombinases are located in the immediate vicinity of the switch and occasionally within the invertible segment (e.g. Hin recombinase in *S*. Typhimurium) [66]. One notable exception is *Bacteroides fragilis* where a single master recombinase of serine-type termed Mpi is responsible for inversion of at least thirteen regions scattered throughout the genome including seven distinct polysaccharide loci [55, 56, 67]. In *C*. *difficile*, the tyrosine-type recombinase RecV is responsible for inversion of *cwpV* (Cdi1) and flagellar (Cdi4) switches [22, 51]. Our results indicate that RecV plays a role in the inversion of four out of five remaining invertible sites. The partial locking of these loci that results upon loss of the RecV recombinase suggests that other recombinases may partner with RecV to mediate inversions of Cdi2, Cdi3 and Cdi5 invertible sites. This is reminiscent of *hin* / *fin* system where two DNA invertases contribute to flagellar phase variation in *S*. Typhimurium [68]. The sequence differences in the inverted repeats further suggest that other recombinases are required. One exception is Cdi2 and Cdi5 which share the same inverted repeats and thus might be inverted by one or more recombinases. Another exception was Cdi7 whose inversion was unaffected by either *recV* inactivation or overexpression (Fig 6). Cdi7 is the only invertible site in *C*. *difficile* strain R20291 genome that features a recombinase in its vicinity. It is therefore possible that inversion of Cdi7 is catalyzed by CDR20291_3416, which is located next to the inversion site. Additional cellular factors or *cis*-acting elements might also participate in the DNA inversion process as was recently demonstrated for the *fim* switch in uropathogenic *E*. *coli* [69].

### Small genomic inversions are widespread in a few bacterial lineages

The method we have developed revealed that DNA inversions are frequently observed in a subset of bacterial species. Out of 44 samples carrying signs of inversions (35 unique species), the majority could be classified as Bacteroidetes, Firmicutes or Proteobacteria phyla. While this distribution probably reflects the uneven composition of our dataset with respect to other phyla rather than biological significance, some interesting patterns nevertheless emerged when positive samples were further reclassified. For example, the vast majority of inversion-positive samples from Firmicutes were members of Clostridia and could be further classified in eight distinct families. On the other hand, only one inversion signature was identified in the Bacilli class despite having substantial number of samples (n = 22, 18 unique species). This result suggests that small genomic inversions were positively selected in the Clostridia versus Bacilli early in the separation of these two lineages. Increasing the sample size and experimental validation of inversion events is necessary to support this hypothesis. Additionally, we cannot exclude that sequencing samples from Bacilli were suboptimal for detection of inversions (inappropriate sequencing library construction, read length, sequencing depth etc.) compared to samples belonging to Clostridia. Similar results were observed in the Proteobacteria phylum where inversion-positives samples were skewed toward the Gammaproteobacteria class. Since sampling size differences were even more pronounced for the Proteobacteria, additional testing in less represented classes such as Alpha-, Beta-, Delta- and Epsilonproteobacteria is needed to fully assess any differences in this lineage. Consistent with reported high prevalence of inversion events in *Bacteroides* species, several members of Bacteroidetes scored positive for inversion signatures in our analysis. Numerous inversion sites were detected in individual genomes of *Bacteroides caccae*, *Odoribacter splanchnicus* and *Parabacteroides distasonis* confirming previous reports in related species [55, 56]. It is believed that *Bacteroides* use genomic inversions to generate subpopulations selectively expressing a wide range of cell surface structures which in turn provides selective advantage for microbes to establish dominance in the ever-challenging colonic environment [56]. Our results suggest that this phenomenon might be applicable to a wider range of species from the Bacteroidetes phylum.

Known examples of genes whose expression is regulated by DNA inversion often revolve around surface components such as flagella, fimbriae, pili, capsule and related surface structures [15–20, 51, 55, 56, 70, 71]. Our analysis suggests that bacteria might use this mechanism to regulate the expression of genes encoding a much larger set of functions than previously observed. For example, genes encoding signaling proteins, which were frequently found in the vicinity of invertible sites in Clostridia species, including the enteric pathogen *C*. *difficile*, solvent-producing *C*. *butyricum* and *C*. *thermocellum*, and environmental isolates *H*. *halobius*, *Desulfosporosinus youngiae* and *Desulfotomaculum gibsoniae*.

In conclusion, our study shows that small genomic inversions, often associated with regulation of expression of neighboring genes, are prevalent in the bacterial world. Some species, including the human opportunistic pathogen *C*. *difficile* seems to have adopted this mode of regulation for a wide variety of functions including cell surface modification, intracellular signaling and environment sensing. As new bacterial strains are sequenced, our method will enable detection of novel inversions potentially providing valuable insights about bacterial evolution and lifestyle.

## Materials and methods

### Bacterial strains and growth conditions

*C*. *difficile* R20291, a ribotype-027 epidemic outbreak strain obtained from Trevor Lawley, was routinely propagated in Brain Hearth Infusion (BHIS) broth supplemented with 0.1% cysteine and 0.5% yeast extract [72]. Cultures were incubated at 37°C in an anaerobic chamber (Coy Laboratories) under anaerobic atmosphere composed of 85% nitrogen, 10% hydrogen and 5% carbon dioxide. *E*. *coli* DH5α and HB101/pK424 were routinely grown in LB broth or agar. Growth media was supplemented with antibiotics when required at following concentrations: ampicillin (100 μg/ml), chloramphenicol (25 μg/ml), thiamphenicol (100 μg/ml) and cycloserine (50 μg/ml).

### Orientation-specific and quantitative PCR assays

For each invertible genomic segment, a set of three primers was designed to amplify published or inverted orientations (see Fig 3A). All primers were designed based on the *C*. *difficile* R20291 published sequence (NCBI Accession No. FN545816.1) and are listed in S1 Table. PCR was carried out on genomic DNA extracted from liquid cultures of three biological replicates grown in BHIS media to a mid-exponential phase (OD_600nm_ ~ 0.4). Similarly, quantitative PCR (qPCR) was used to evaluate the proportion of the bacterial population that was in either published or inverted orientation at mid-exponential growth phase (OD_600nm_ ~ 0.4) and also early stationary phase (~ 2h after entering in stationary phase of growth). Amplifications were carried out in a Mx3005P qPCR system (Stratagene) in a total volume of 20 μl with the following components: 1X PCR buffer (12 mM Tris-HCl, pH 8.3, 50 mM KCl, 8 mM MgCl_2_, 150mM trehalose, 0.2% Tween 20, 0.2 mg/ml bovine serum albumin, 0.2X SYBR green I (Invitrogen)), 0.5 units of *Taq* DNA polymerase (New England BioLabs), 200 ng of genomic DNA and one of the primer sets specific for either the published or inverted orientation for Cdi1-6. For the Cdi7 inversion site, iTaq Universal SYBR Green Supermix (BioRad) was used following the manufacturer’s recommendations. The following cycling conditions were used: 95°C for 2 min, followed by 40 cycles of 95°C for 15 s and 60°C for 1 min. Detailed primer concentrations, amplification efficiencies and amplicons lengths are summarized in S3 Table. The ΔΔ*C*_*T*_ method was used to calculate the ratio of published versus inverted configurations with *rpoA* as internal reference control as described in S2 Methods.

### SNAP-tag reporter constructions

Previously described *codA*-based allelic exchange method [73] was used to replace CDR20291_3128 coding sequence (GenBank accession: CBE06969.1) with the *C*. *difficile* codon-optimized SNAP-tag coding sequence. Briefly, approximately 1300 bp genomic fragments were amplified upstream (primers OS158 and OS159) and downstream (primers OS162 and OS163) of the start and stop codons of CDR20291_3128. A *C*. *difficile* codon-optimized SNAP-tag coding sequence was amplified from pFT46 vector using primers OS160 and OS161 [74]. Complementary overlapping sequences were added to the 5-prime end of primers to allow for accurate fusion of all PCR products into *Pme*I-linearized pMTL-SC7315 vector using Gibson Assembly Master Mix (New England BioLabs). The resulting plasmid was then conjugated into *C*. *difficile* R20291 strain using the heat-stimulated conjugation method described elsewhere [75]. Mutants were selected as previously described [73] and screened by colony-PCR for the presence of the right SNAP-chromosomal junction and the absence of CDR20291_3128 coding sequence. All PCR products were sequenced to confirm the genetic construct and the absence of any secondary mutations. Additionally, Illumina sequencing of whole-genome DNA revealed no off-target mutations, and the genetic background was otherwise identical to the parental R20291 aside from the anticipated SNAP-tag allelic replacement. One representative clone was selected for fluorescent labeling.

### Fluorescence microscopy

Fluorescent labeling of *C*. *difficile* cells was done as previously described with modifications [76]. Briefly, 5 ml of exponentially growing cultures (OD_600nm_ ~ 0.4) were washed once in PBS and resuspended in 0.1 ml PBS containing 250 nM SNAP-Cell TMR-Star (New England BioLabs) for 30 min at 37°C. The excess of fluorescent substrate was removed by washing the cells 5 times with PBS prior to mounting on glass slides with freshly prepared 1% agarose pads. Imaging was done on Eclipse 80*i* fluorescence microscope (Nikon) at 60X magnification using Photometrics CoolSNAP HQ camera (Roper Scientific) operated by NIS-Elements software (Nikon). Acquired images were further minimally processed and pseudocolored in Photoshop CC 2018 (Adobe Systems). Cells showing uniform fluorescence in the red channel were counted and compared to the total number of cells observed in the bright field. Counts were done on at least 5 images from different fields on two biological replicates.

### Overexpression of RecV

*recV* (CDR20291_1004) was cloned in pRPF185 vector using SacI and BamHI restriction sites as previously described [51]. A control plasmid (empty vector) of the ATc-inducible expression vector was generated by removing the *gusA* gene from pRPF185 by digestion with SacI and BamHI and religating the vector backbone following treatment with Klenow. *C*. *difficile* strain R20291 containing pRPF185 empty vector and pRPF185:*recV* were grown overnight in Tryptone-Yeast Extract (TY) broth supplemented with thiamphenicol. The following day, cultures were back-diluted 1:50 into TY + thiamphenicol and grown to OD_600nm_ = 0.3. Once the cultures reached the right optical density, anhydrotetracycline (20 ng/ml) was added and cultures were further incubated until early stationary phase was (OD_600nm_ ~ 1.5) at which point cells were pelleted and genomic DNA extracted by phenol-chloroform as previously described. Quantitative PCR was performed with SensiMix SYBR (BioLine) with 100 ng of genomic DNA and 100 nM primers in 20 μl. Reactions were run on Lightcycler 96 system (Roche) with the following three-step cycling conditions: 98°C for 2 min, followed by 40 cycles of 98°C for 30 s and 60°C for 1 min and 72°C for 30 sec. Quantification was done with ΔΔ*C*_*T*_ with *rpoA* as a reference as previously described.

### Short sequence alignments and bioinformatics analysis

Random DNA sequence was generated using Bioinformatics Toolbox from Matlab R2017b (MathWorks). Paired-end Illumina high-throughput sequencing reads were simulated using ART-MountRainier-2016-06-05 [49] using Illumina HiSeq 2500 and HiSeq 2000 built-in parameters with read length of 50, 100 or 150 bp and mean fragment size of 250, 500 or 800 bp ± 20% standard deviation. Read alignments were done using bwa version 0.7.13-r1126 [77]. Template size was inferred from bwa mappings using a custom script. SAM file manipulation was done using SAMtools version 1.5 [78]. Sequencing coverage was calculated using bedtools v2.26.0 [79] and HTSeq version 0.9.1 [80]. Extraction of same-orientation reads and 5-prime soft-clipped reads from SAM files was done using custom scripts as explained in S1 Methods. All scripts are available from https://github.com/camillilab/analyze_clusters. Some analyses were conducted in *R* version 3.4.1 [81]. Protein domain analysis was carried out using InterPro database (https://www.ebi.ac.uk/interpro/) [82]. Terminal inverted repeats were identified by pairwise sequence alignment of invertible site boundaries using EMBOSS Water hosted on The European Bioinformatics Institute’s (EMBL) website (https://www.ebi.ac.uk/Tools/psa/emboss_water/nucleotide.html).

## Supporting information

S1 Fig *In silico* validation of inversion detection. (A) Five distinct clusters (black circles) composed of unidirectional read pairs are detected from the simulated sequencing dataset. The size of the introduced inversion is given above while the genomic position is given beneath the clusters. (B) Same-orientation reads enrichment for the 500 bp inversion. (C) 5-prime end trimmed reads enrichment for the 500 bp inversion.
(TIF)

S2 Fig Read length and insert size affects recovery of same-orientation reads. Influence of read length (50, 100 and 150 bp) and insert-size (250, 500 and 800 bp) was analyzed on the inversion detection efficiencies as assessed by the number of same-orientation read counts recovered for different inversion sizes (100, 250, 500, 1000 and 5000 bp) and proportions (0.01, 0.1, 1, 10 and 50%). Simulation details, data acquisition and analysis are described in S1 Methods.
(TIF)

S3 Fig Sequencing depth affects recovery of same-orientation reads. Total same-orientation read counts are reported on the *x* axis as determined from simulated sequencing datasets with variable number of total read counts reported on the *y* axis. Simulated read length was 100 bp with an average insert size of 500 ± 100 bp for all datasets.
(TIF)

S4 Fig Transcriptome analysis suggests CDR20291_3126–28 are part of an operon. Open reading frames identified by their respective locus_tag number are illustrated by the gray arrowed boxes. Transcriptional profile from *C*. *difficile* R20291 grown in TY medium to mid-exponential growth phase [53] is illustrated by the orange line. Read counts are indicated with the scale on the left. CDR20291_3126–3128 have different expression levels compared to surrounding genes suggesting a possible operon structure.
(TIF)

S1 Table Primers used in this study. (DOCX)

S2 Table Conservative site-specific recombination detection among a set of bacterial and archaeal samples with publicly available paired-end sequencing datasets. (XLSX)

S3 Table qPCR primers used in this study. (DOCX)

S1 Methods *In silico* and bioinformatics analysis. (DOCX)

S2 Methods Quantification of inversion frequencies using ΔΔCt method. (DOCX)
