# Supplementary figures and images for: Genome-wide detection of conservative site-specific recombination in bacteria
Source: PLoS Genet. 2018 Apr 5;14(4):e1007332. doi: 10.1371/journal.pgen.1007332 (PMC5903667; doi:10.1371/journal.pgen.1007332)

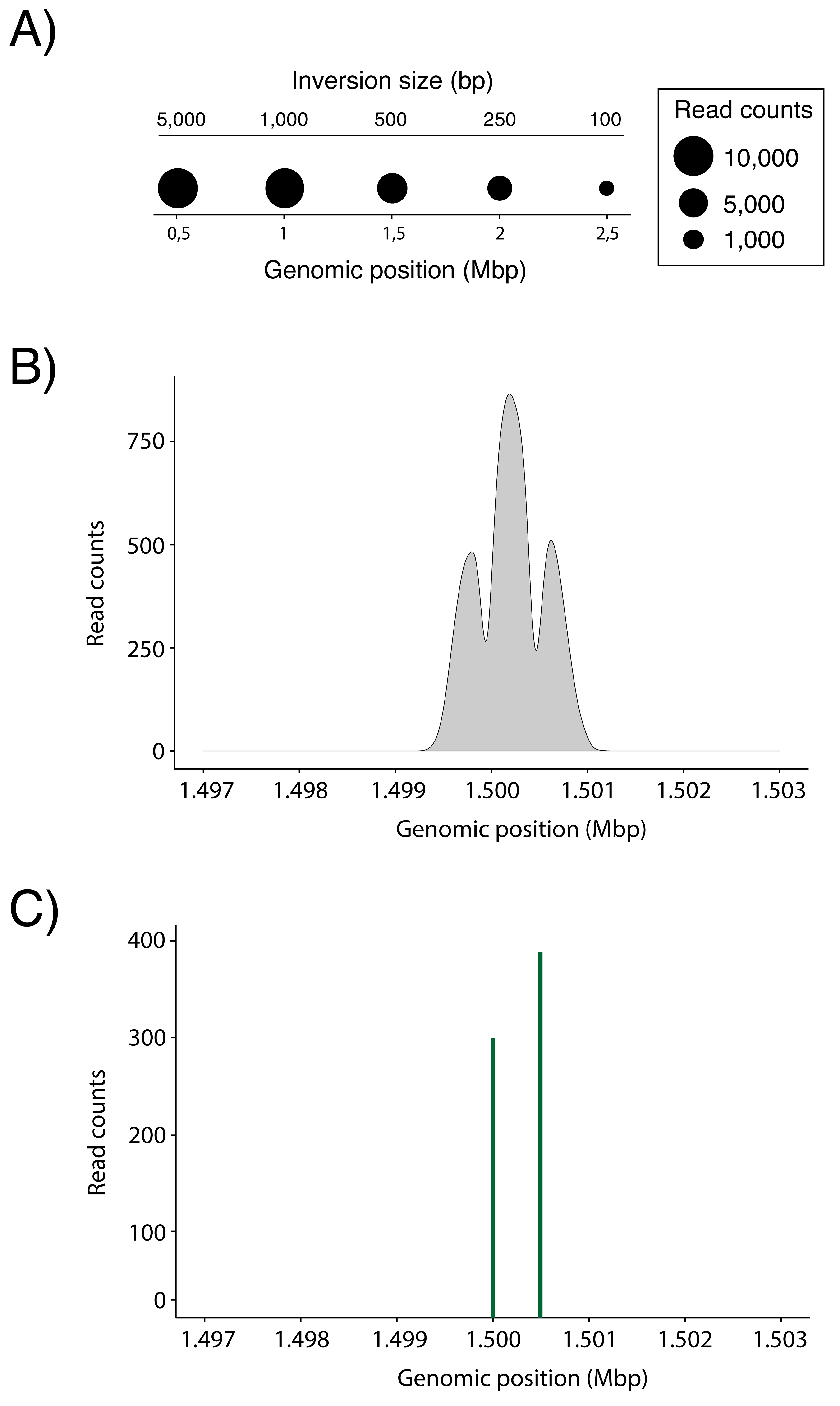

Supplement: S1 Fig — (A) Five distinct clusters (black circles) composed of unidirectional read pairs are detected from the simulated sequencing dataset. The size of the introduced inversion is given above while the genomic position is given beneath the clusters. (B) Same-orientation reads enrichment for the 500 bp inversion. (C) 5-prime end trimmed reads enrichment for the 500 bp inversion. (TIF) [file pgen.1007332.s001.tif]

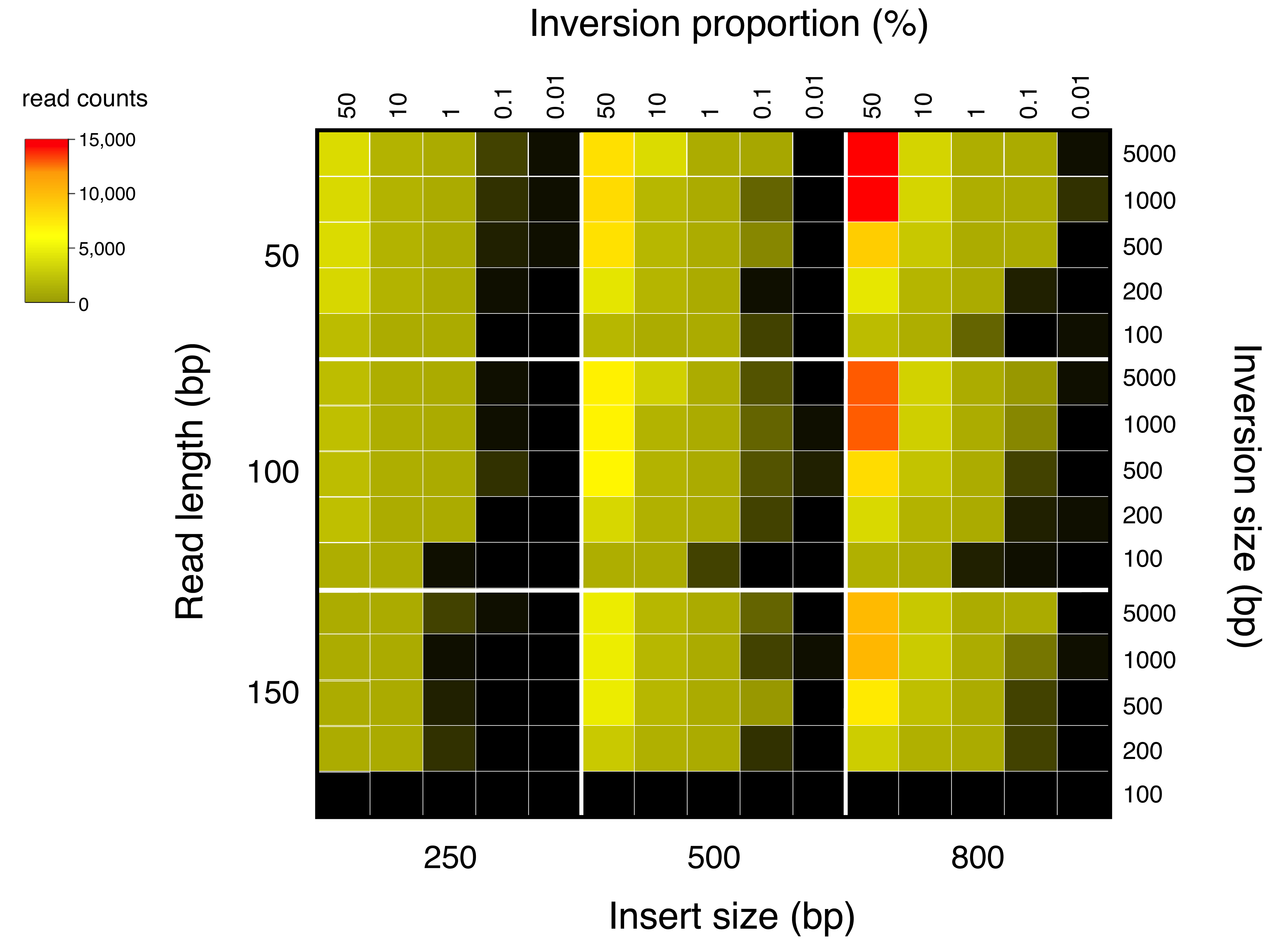

Supplement: S2 Fig — Influence of read length (50, 100 and 150 bp) and insert-size (250, 500 and 800 bp) was analyzed on the inversion detection efficiencies as assessed by the number of same-orientation read counts recovered for different inversion sizes (100, 250, 500, 1000 and 5000 bp) and proportions (0.01, 0.1, 1, 10 and 50%). Simulation details, data acquisition and analysis are described in S1 Methods. (TIF) [file pgen.1007332.s002.tif]

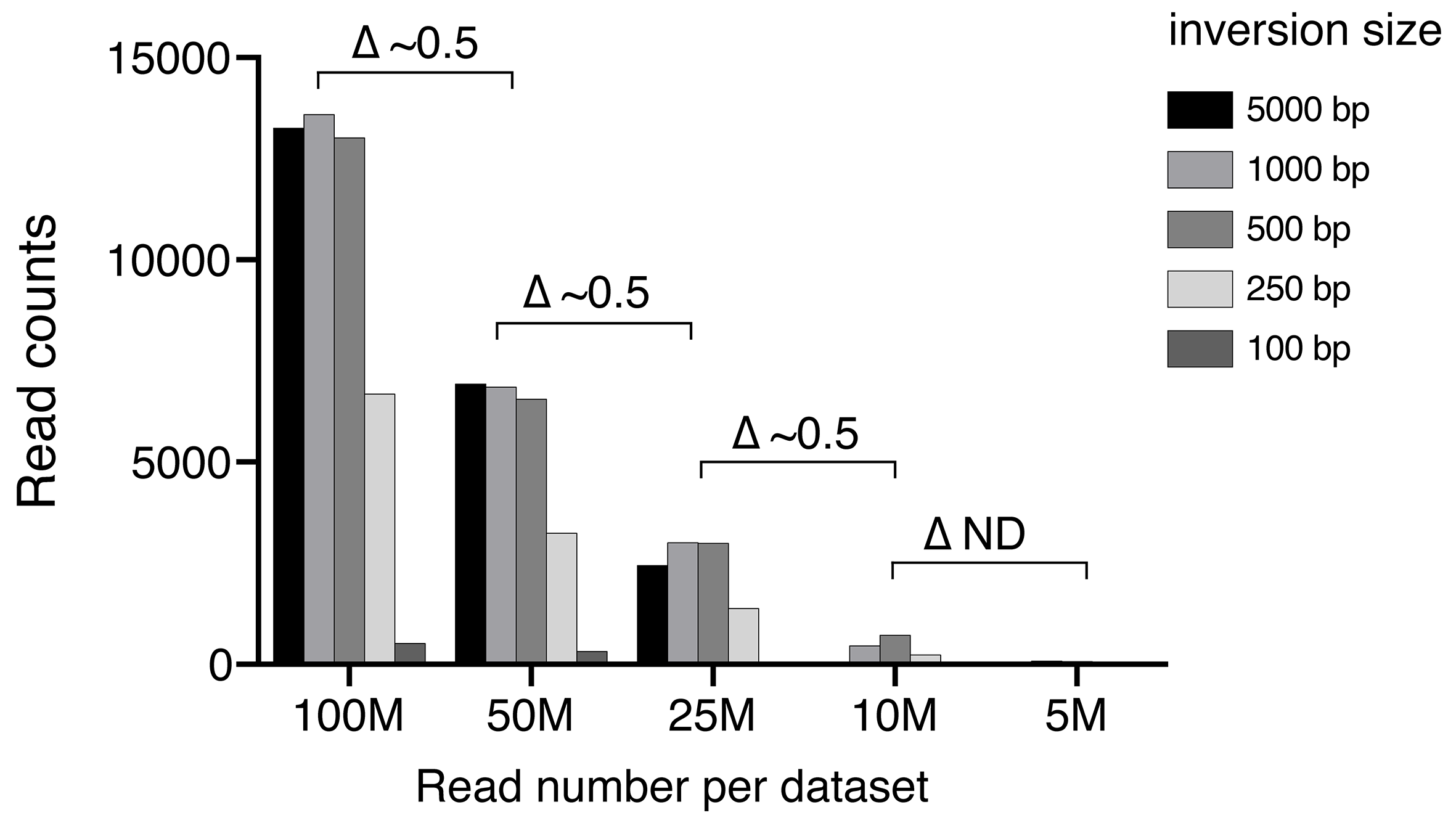

Supplement: S3 Fig — Total same-orientation read counts are reported on the x axis as determined from simulated sequencing datasets with variable number of total read counts reported on the y axis. Simulated read length was 100 bp with an average insert size of 500 ± 100 bp for all datasets. (TIF) [file pgen.1007332.s003.tif]

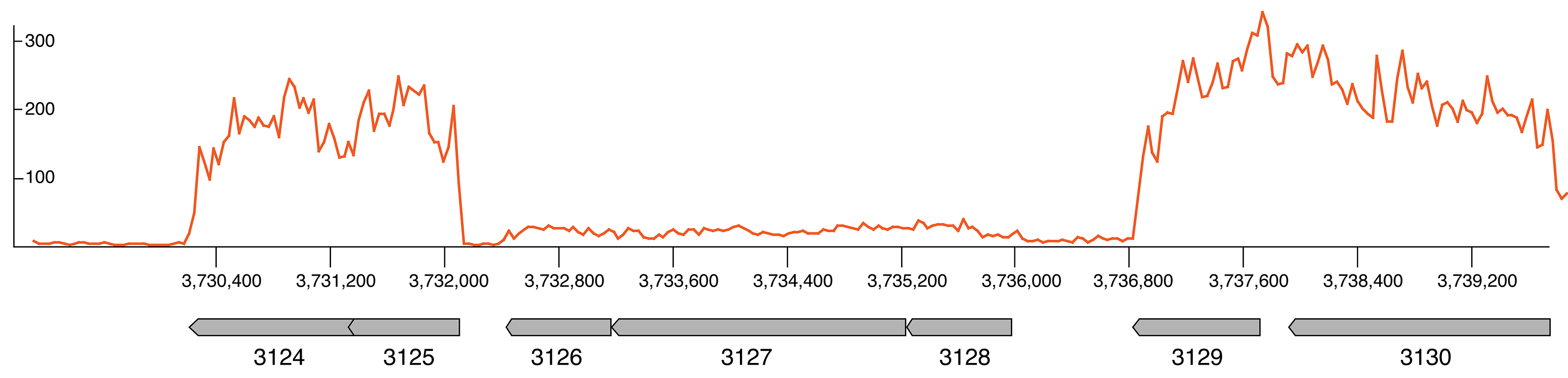

Supplement: S4 Fig — Open reading frames identified by their respective locus_tag number are illustrated by the gray arrowed boxes. Transcriptional profile from C. difficile R20291 grown in TY medium to mid-exponential growth phase [53] is illustrated by the orange line. Read counts are indicated with the scale on the left. CDR20291_3126–3128 have different expression levels compared to surrounding genes suggesting a possible operon structure. (TIF) [file pgen.1007332.s004.tif]
