# Supplementary material for: Genome-wide detection of conservative site-specific recombination in bacteria
Source: PLoS Genet. 2018 Apr 5;14(4):e1007332. doi: 10.1371/journal.pgen.1007332 (PMC5903667; doi:10.1371/journal.pgen.1007332)
Supplement: S1 Table — (DOCX) [file pgen.1007332.s005.docx]

**S1 Table. Primers used in this study.**

| **Primer** | **Target*** | **Strand** | **Sequence (5’- 3’)** |
| --- | --- | --- | --- |
| RT1920 | Cdi1 OFF state | Fwd | CAAAACCATGTTTTTTATAACAATTCATTAAC |
| RT1050 |  | Rev | AGCATCTGCTATAGATGAGTCGTTT |
| RT1921 | Cdi1 ON state | Rev | GTTAATGAATTGTTATAAAAAACATGGTTTTG |
| RT1050 |  | Rev | AGCATCTGCTATAGATGAGTCGTTT |
| OS100 | Cdi2 PUB state | Fwd | GTTAAAAATTTAAGATATCTTTTCAGTATAATGGA |
| RT2195 |  | Rev | TTTTGTTTTTATGTTAGGATATTTCTTAGAAATG |
| OS100 | Cdi2 INV state | Fwd | GTTAAAAATTTAAGATATCTTTTCAGTATAATGGA |
| OS101 |  | Fwd | CATTTCTAAGAAATATCCTAACATAAAAACAAAA |
| RT1455 | Cdi3 INV state | Fwd | CAAGAATTCCAAGTTGAAAGTGAGATAC |
| RT1737 |  | Fwd | CAGACAATTCAAACAAAAATAATC |
| RT1455 | Cdi3 PUB state | Fwd | CAAGAATTCCAAGTTGAAAGTGAGATAC |
| RT1738 |  | Rev | GATTATTTTTGTTTGAATTGTCTG |
| RT1614 | Cdi4 ON state | Fwd | AGGCAACTTTATAAAGAAATATTTAAATTTATATTAAAATATTTTTATATTTTTATTAGG |
| RT857 |  | Rev | AGGCATAGCATCATTTAATGTTTCTTC |
| RT1615 | Cdi4 OFF state | Rev | CCTAATAAAAATATAAAAATATTTTAATATAAATTTAAATATTTCTTTATAAAGTTGCCT |
| RT857 |  | Rev | AGGCATAGCATCATTTAGTGTTTCTTC |
| OS103 | Cdi5 PUB state | Fwd | CTACTGAATATCATATTAGACTCCTTTCTTAACCA |
| OS104 |  | Rev | GTAAATTAAGATGTATTTCATTTCTCAAAAATATCCT |
| OS103 | Cdi5 INV state | Fwd | CTACTGAATATCATATTAGACTCCTTTCTTAACCA |
| RT2196 |  | Fwd | AGGATATTTTTGAGAAATGAAATACATCTTAATTTAC |
| OS109 | Cdi6 PUB state | Rev | GGAGATATATGGAGTTAGTGGTGCAA |
| RT2197 |  | Fwd | TATTTTTCTAGAAACTTGTCTATTGGCTAG |
| OS109 | Cdi6 INV state | Rev | GGAGATATATGGAGTTAGTGGTGCAA |
| OS110 |  | Rev | CTAGCCAATAGACAAGTTTCTAGAAAAATA |
| OS196 | Cdi7 PUB state | Fwd | GTACAGAAGTTACCCAGAAGCTTGT |
| OS197 |  | Rev | TCCCCGCAATGGATGTTTTTTAATTCATC |
| OS196 | Cdi7 INV state | Fwd | GTACAGAAGTTACCCAGAAGCTTGT |
| OS198 |  | Fwd | TCCCAATTTAAATGTAGAGGTCATCAAT |
| OS158 | 3128::SNAP,  left-side homology | Rev | GTGTTTTTTGTTACCCTAAGTTTAGTAATAGTATCAAGAGAAGAAG |
| OS159 |  | Fwd | CTTTATCCATTACACCACTCCATTCAAAG |
| OS162 | 3128::SNAP,  right-side homology | Rev | ACTTGGGTAAGTGAGATACTGTAATTAATAAATAGTTCTTG |
| OS163 |  | Fwd | AGATTATCAAAAAGGAGTTTCCACATCTGCCAAGAATTTTTTAC |
| OS160 | 3128::SNAP,  SNAP-tag | Fwd | GAGTGGTGTAATGGATAAAGATTGTGAAATGAAG |
| OS161 |  | Rev | AGTATCTCACTTACCCAAGTCCTGGTTTC |
| OS175 | 3128::SNAP,  in SNAP-tag | Fwd | TGGTGAAGAGCTGGAACCGGA |
| OS178 | 3128::SNAP,  in the genome | Rev | TGGGCATCTGGAGATATATGGAGTTAG |

Notes:

*INV refers to the inverted state of the switch; PUB refers to the published orientation of the switch
