# Supplementary material for: Genome-wide detection of conservative site-specific recombination in bacteria
Source: PLoS Genet. 2018 Apr 5;14(4):e1007332. doi: 10.1371/journal.pgen.1007332 (PMC5903667; doi:10.1371/journal.pgen.1007332)
Supplement: S3 Table — (DOCX) [file pgen.1007332.s007.docx]

**S3 Table. qPCR primers used in this study.**

| **Primer** | **Target** | **Strand** | **Primer**  **concentration**  **(nM)** | **Sequence (5’- 3’)** | **Efficiency of amplification**  **(%)** |
| --- | --- | --- | --- | --- | --- |
| OS138 | Cdi1 OFF state | Fwd | 100 | CGCAATTATTTGTTTTTCATATGGATAAAATTGG | 96 |
| OS139 |  | Rev | 100 | GATTTTTATGTTAATGAATTGTTATAAAAAACATGG |  |
| OS140 | Cdi1 ON state | Fwd | 200 | GGTAAGTTTGATTTTTATGTTAATGAATTG | 98 |
| OS141 |  | Fwd | 200 | CAGTTTGTGCACTAGCTATGCCTGC |  |
| OS101 | Cdi2 PUB state | Fwd | 100 | CATTTCTAAGAAATATCCTAACATAAAAACAAAA | 95 |
| OS134 |  | Rev | 100 | CGATTACACTACAGAATTAGAATGTCAATG |  |
| OS100 | Cdi2 INV state | Fwd | 100 | GTTAAAAATTTAAGATATCTTTTCAGTATAATGGA | 96 |
| OS101 |  | Fwd | 100 | CATTTCTAAGAAATATCCTAACATAAAAACAAAA |  |
| OS106 | Cdi3 INV state | Fwd | 100 | GATTTGTCGAAACCATTGTAATAAGA | 100 |
| OS107 |  | Fwd | 100 | CAATAGTTAAGACAATGAATATGCTACATTCT |  |
| OS107 | Cdi3 PUB state | Fwd | 100 | CAATAGTTAAGACAATGAATATGCTACATTCT | 93 |
| OS136 |  | Rev | 100 | GTAAATTCCTCATAAAAATTTCCTCCCA |  |
| OS97 | Cdi4 ON state | Fwd | 200 | GTTGCCAAAAAGAGTTCTAGGTATATTGTATA | 99 |
| OS98 |  | Fwd | 200 | GGATTTACGAAAATAATATTAGTTTTCTTACC |  |
| OS98 | Cdi4 OFF state | Fwd | 100 | GGATTTACGAAAATAATATTAGTTTTCTTACC | 100 |
| OS133 |  | Rev | 100 | TGTCATATGTATTCACACTCACTCCT |  |
| OS104 | Cdi5 PUB state | Rev | 100 | GTAAATTAAGATGTATTTCATTTCTCAAAAATATCCT | 97 |
| OS135 |  | Fwd | 100 | GCTTTTATCGCAAGTTTGTTTTAAATGAC |  |
| OS104 | Cdi5 INV state | Rev | 100 | GTAAATTAAGATGTATTTCATTTCTCAAAAATATCCT | 95 |
| OS105 |  | Rev | 100 | GTAAAGTTTATAAAATCTGAAAAGCTCAAGA |  |
| OS110 | Cdi6 PUB state | Rev | 100 | CTAGCCAATAGACAAGTTTCTAGAAAAATA | 98 |
| OS137 |  | Fwd | 100 | GAACAATTCTTGAATATTGTATTGAACATTAAGA |  |
| OS109 | Cdi6 INV state | Rev | 100 | GGAGATATATGGAGTTAGTGGTGCAA | 95 |
| OS110 |  | Rev | 100 | CTAGCCAATAGACAAGTTTCTAGAAAAATA |  |
| OS196 | Cdi7 PUB state | Fwd | 300 | GTACAGAAGTTACCCAGAAGCTTGT | 98 |
| OS197 |  | Rev | 300 | TCCCCGCAATGGATGTTTTTTAATTCATC |  |
| OS196 | Cdi7 INV state | Fwd | 300 | GTACAGAAGTTACCCAGAAGCTTGT | 96 |
| OS198 |  | Fwd | 300 | TCCCAATTTAAATGTAGAGGTCATCAAT |  |
| OS142 | *rpoA* | Fwd | 100 | TCATTACCAGGTGTAGCAGTGAATGC | 99 |
| OS143 |  | Rev | 100 | TGATAGAGCATGGTCCTTGAGCTTCT |  |
