# Supplementary material for: Genome-wide detection of conservative site-specific recombination in bacteria
Source: PLoS Genet. 2018 Apr 5;14(4):e1007332. doi: 10.1371/journal.pgen.1007332 (PMC5903667; doi:10.1371/journal.pgen.1007332)
Supplement: S1 Methods — (DOCX) [file pgen.1007332.s008.docx]

**S1 Methods**

***in silico* random sequence generation**

A random DNA sequence with the length of 4,659,625 bp was created using the Bioinformatics toolbox from MathWorks MATLAB R2017b (9.3.0.713579) with the following command lines:

> RandomSeq = randseq(4659625)

> fastawrite('name','header', RandomSeq);

Genomic inversions of 100, 250, 500, 1000 and 5000 base pairs were introduced manually by reverse-complementing the target sequence with the reverse-complement tool at <https://www.bioinformatics.org/sms/rev_comp.html> and accordingly modifying the backbone random sequence with CLC Main Workbench Version 7.9.1. Paired-end Illumina high-throughput sequencing reads were simulated from both modified and unmodified random sequences using ART-MountRainier-2016-06-05 with the following generic command line:

> art_illumina -ss HS25 -i RandomSeq.fasta -p -na -c 50000000 -l 100 -m 500 -s 100 -o RandomSeq_out

where -ss HS25 specifies HiSeq2500 as the Illumina sequencing system of the built-in profile used for simulation, -i RandomSeq.fasta indicates the fasta file to use as reference, -p indicate a paired-end read simulation, -na instructs to not output the alignment file,
-c 5000000 indicates the total number of reads (50 million) to output, -l 100 indicates read length of 100 bp, -m 500 indicates a mean insert size of 500 bp, -s 100 indicates the standard deviation of DNA fragment size, -o RandomSeq_out indicates the prefix name for the output fastq file.

When needed, fastq files where combined together using the following command line:

> cat RandomSeq_1.fq RandomSeq_2.fq > RandomSeq.fq

**Illumina short-read alignment, same-orientation and 5-prime end clipped read extraction**

Short read sequences were aligned using bwa with following generic command lines:

> bwa index -p INDEX reference.fasta

> bwa aln INDEX file_1.fastq > file_1.sai

> bwa aln INDEX file_2.fastq > file_2.sai

> bwa sampe INDEX file_1.sai file_2.sai file_1.fastq file_2.fastq | samtools view -q 1 > aln.sam

Same orientation reads were extracted with the following command line:

> awk '$2 ~ /113|177|65|129/ {print $2, $4, $6, $9}' infile.sam > same_orientation_reads.txt

5-primed end clipped reads were extracted using the following command line:

> awk '($2 ~ /147|83/ && $6 ~ /^..?S/) || ($2 ~ /99|163/ && $6 ~ /S$/) {next;} $6 ~ /^..?S/ {print $2, $4, $6, $9 }' infile.sam > 5_prime_clipped_reads.txt
