# Supplementary material for: Genome-wide detection of conservative site-specific recombination in bacteria
Source: PLoS Genet. 2018 Apr 5;14(4):e1007332. doi: 10.1371/journal.pgen.1007332 (PMC5903667; doi:10.1371/journal.pgen.1007332)
Supplement: S2 Methods — (DOCX) [file pgen.1007332.s009.docx]

**S2 Methods**

**Quantification of inversion frequencies using ∆∆Ct method**

The standard ∆∆Ct method can be used to assess gene expression levels from two different and independent conditions, i.e. X is a gene dosage in some reference condition and Y is a dosage of the same gene in some altered condition. The fold change of expression can be calculated with the following equation:

$FD= 2^{-\Delta\Delta Ct}$ , where $\Delta\Delta Ct=\left( {Ct}_{Y}- {Ct}_{Y-REF} \right)-({Ct}_{X}-{Ct}_{X-REF})$

For the relative quantification of two distinct states (ex. reference “REF” and inverted “INV”) of a single genomic region, the equation has to be adjusted because REF and INV are dependent and mutually exclusive, i.e. if INV dosage increases, REF dosage decreases proportionally and vice versa. Accordingly, INV is not compared to REF but rather to REF-INV.
If: REF = proportion (%) of reference state, INV = proportion (%) of the inverted state and FD = fold change as calculated by the equation above, thus:

$REF+INV=100\%, \mathrm{therefore} INV=100-REF$ (1)

$FD= \frac{INV}{REF}, therefore INV= FD*REF$ (2)

Substitute equation 1 into equation 2: $100-REF=FD*REF$

Isolate FD by dividing both sides with REF:

$\frac{100-REF}{REF}=\frac{FD*REF}{REF}$ (3)

Resolve equation 3: $\frac{100}{REF}-\frac{REF}{REF}=FD$, thus:

$FD=\frac{100}{REF}-1$ , isolate REF: (4)

$REF= \frac{100}{FD+1}$ (5)

Equation 5 can be used to correct the percentage of reference state. Inverted state can then be calculated using equation 1.
